# Airway metabolic profiling during *Streptococcus pneumoniae* infection identifies branched chain amino acids as signatures of upper airway colonisation

**DOI:** 10.1371/journal.ppat.1011630

**Published:** 2023-09-05

**Authors:** Angharad E. Green, Sian Pottenger, Manal S. Monshi, Thomas E. Barton, Marie Phelan, Daniel R. Neill

**Affiliations:** 1 Department of Clinical Infection, Microbiology and Immunology, Institute of Infection, Veterinary and Ecological Sciences, University of Liverpool, Liverpool, United Kingdom; 2 Highfield NMR Facility, Liverpool Shared Research Facilities (LIV-SRF), University of Liverpool, Liverpool, United Kingdom; 3 Department of Biochemistry and Systems Biology, Institute of Molecular, Systems and Integrative Biology, University of Liverpool, Liverpool, United Kingdom; The University of Alabama at Birmingham, UNITED STATES

## Abstract

*Streptococcus pneumoniae* is a leading cause of community-acquired pneumonia and bacteraemia and is capable of remarkable phenotypic plasticity, responding rapidly to environmental change. Pneumococcus is a nasopharyngeal commensal, but is responsible for severe, acute infections following dissemination within-host. Pneumococcus is adept at utilising host resources, but the airways are compartmentalised and those resources are not evenly distributed. Challenges and opportunities in metabolite acquisition within different airway niches may contribute to the commensal-pathogen switch when pneumococcus moves from nasopharynx into lungs. We used NMR to characterise the metabolic landscape of the mouse airways, in health and during infection. Using paired nasopharynx and lung samples from naïve animals, we identified fundamental differences in metabolite bioavailability between airway niches. Pneumococcal pneumonia was associated with rapid and dramatic shifts in the lung metabolic environment, whilst nasopharyngeal carriage led to only modest change in upper airway metabolite profiles. NMR spectra derived from the nasopharynx of mice infected with closely-related pneumococcal strains that differ in their colonisation potential could be distinguished from one another using multivariate dimensionality reduction methods. The resulting models highlighted that increased branched-chain amino acid (BCAA) bioavailability in nasopharynx is a feature of infection with the high colonisation potential strain. Subsequent analysis revealed increased expression of BCAA transport genes and increased intracellular concentrations of BCAA in that same strain. Movement from upper to lower airway environments is associated with shifting challenges in metabolic resource allocation for pneumococci. Efficient biosynthesis, liberation or acquisition of BCAA is a feature of adaptation to nasopharyngeal colonisation.

## Author summary

Several species of bacterial pathogens that cause disease in humans are often found residing in the upper reaches of the airways. Within this environment, they rarely cause ill health, but they can be responsible for severe disease when they reach the lower regions of the respiratory tract, such as the lungs. The reasons why the same bacteria can be benign in one airway region and pathogenic in another are poorly understood. In this study, we defined the chemical environment of different airway regions, to determine what resources were available for bacteria to use for metabolism and growth. We identified gradients of chemical abundance within the airways, including in a group of amino acids (the building blocks of proteins) that trigger important changes in bacteria that influence their disease-causing potential. Using a pathogenic bacterial species–*Streptococcus pneumoniae–*we show that the ability of the bacteria to produce or acquire those same amino acids determines how successfully they can colonise the airways. Defining airway environments, and the challenges and opportunities posed to bacteria that colonise them, will help us understand the relationship between asymptomatic airway infection and diseases such as pneumonia.

## Introduction

*Streptococcus pneumoniae* is an opportunistic pathogen, capable of causing localised infections in the airways and middle ear, as well as serious systemic infections. The primary niche of pneumococcus is the human nasopharynx, within which it establishes a commensal-like lifestyle that is ordinarily associated with only subclinical inflammation. Many host and pathogen factors contributing to infection outcomes during pneumococcal colonisation and disease have been described [1], but the principal drivers of the commensal-pathogen switch remain elusive. What is clear, is that the two-way dialogue taking place at the host-pathogen interface can profoundly influence the course of infection. Host detection of pathogen-associated molecular patterns is required for induction of innate immune responses, which can be protective or pathological [2]. Equally important, however, is bacterial sensing of the host environment, as exemplified by the niche-specific gene expression signatures of pneumococci [3].

Host factors act as chemical cues, triggering pneumococcal signalling systems that control bacterial metabolism, virulence and antimicrobial resistance [4–6]. The host is a rich source of carbohydrates, on which pneumococci are reliant for growth [7]. To liberate sugars from the branched mucin glycans of the airways, pneumococci produce an array of glycosyl hydrolases, expression of which is under tight environment-specific regulation [8–10]. Amino acid metabolism is equally critical to survival within-host and is similarly environmentally regulated, both in pneumococci and in other opportunistic pathogens [11,12]. The metabolic flexibility of pneumococcus is achieved through the actions of a set of master nutritional regulatory proteins, principally CodY, CcpA and GlnR [13–17]. These master regulators, in turn, control the activities of other regulatory systems, including the Rgg/Shp and TprA/PhrA quorum sensing systems, through which they indirectly influence broad regulons [18,19]. The contributions of these systems to pneumococcal colonisation and disease have been elegantly defined in recent years, but it is not clear what the *in vivo* triggers for their activation or repression might be. This is due, in part, to the limited information available on the metabolic profiles of the various host niches inhabited by pneumococci.

Here, we use NMR metabolomics to define the nutritional environments of nasopharynx, as the primary colonisation site of the pneumococcus, and lungs, a key niche within which disease can develop. We undertook these studies using mice, allowing us to take matched nasopharynx and lung samples from a single animal, and to study both the impact of pneumococcal upper airway colonisation, and of lung infection, on metabolite bioavailability in the respiratory tract. Our results highlight the compartmentalisation of the airways, with substantial differences in the metabolic landscapes of nasopharynx and lungs, and niche-specific influences of bacterial infection on metabolic profiles. Regional differences in airway metabolites may contribute chemical cues that lead to phenotypic change in pneumococci. We identify branched-chain amino acid (BCAA) bioavailability in the nasopharynx as a correlate of pneumococcal colonisation potential. These data will inform future studies aiming to determine the nutritional cues that contribute to the commensal-pathogen switch in pneumococci.

## Results

### Nasopharynx and lungs are metabolically distinct airway environments

**Fig 1:**
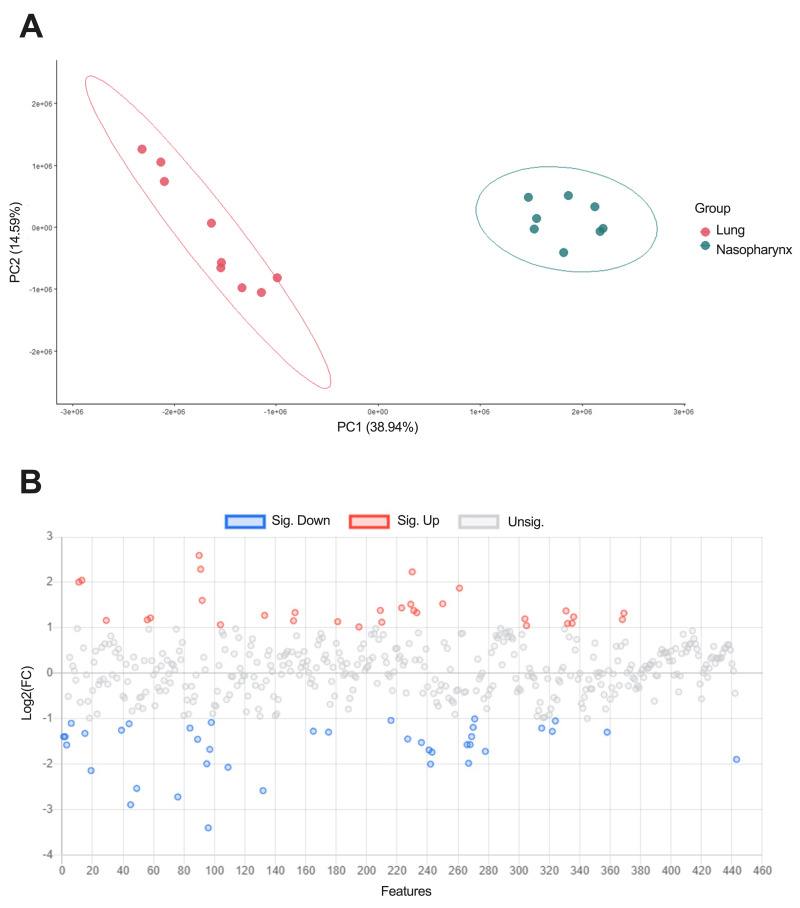
Murine lung and nasopharynx have distinct metabolic profiles. **A.** Principal component analysis of NMR metabolomics data from paired nasopharynx and lungs samples from uninfected mice, after normalisation by PQN. Tissue samples that failed NMR QC checks were excluded. N = 9 for lung and N = 8 for nasopharynx. PCA plot ellipses indicate a 95% confidence level. **B.** Relative abundance of individual metabolites, annotated NMR spectra peaks, in lungs vs nasopharynx. Metabolites shown in red are those more abundant in lung (>1 Log2 fold-change), those in blue are more abundant in nasopharynx.

**Fig 2:**
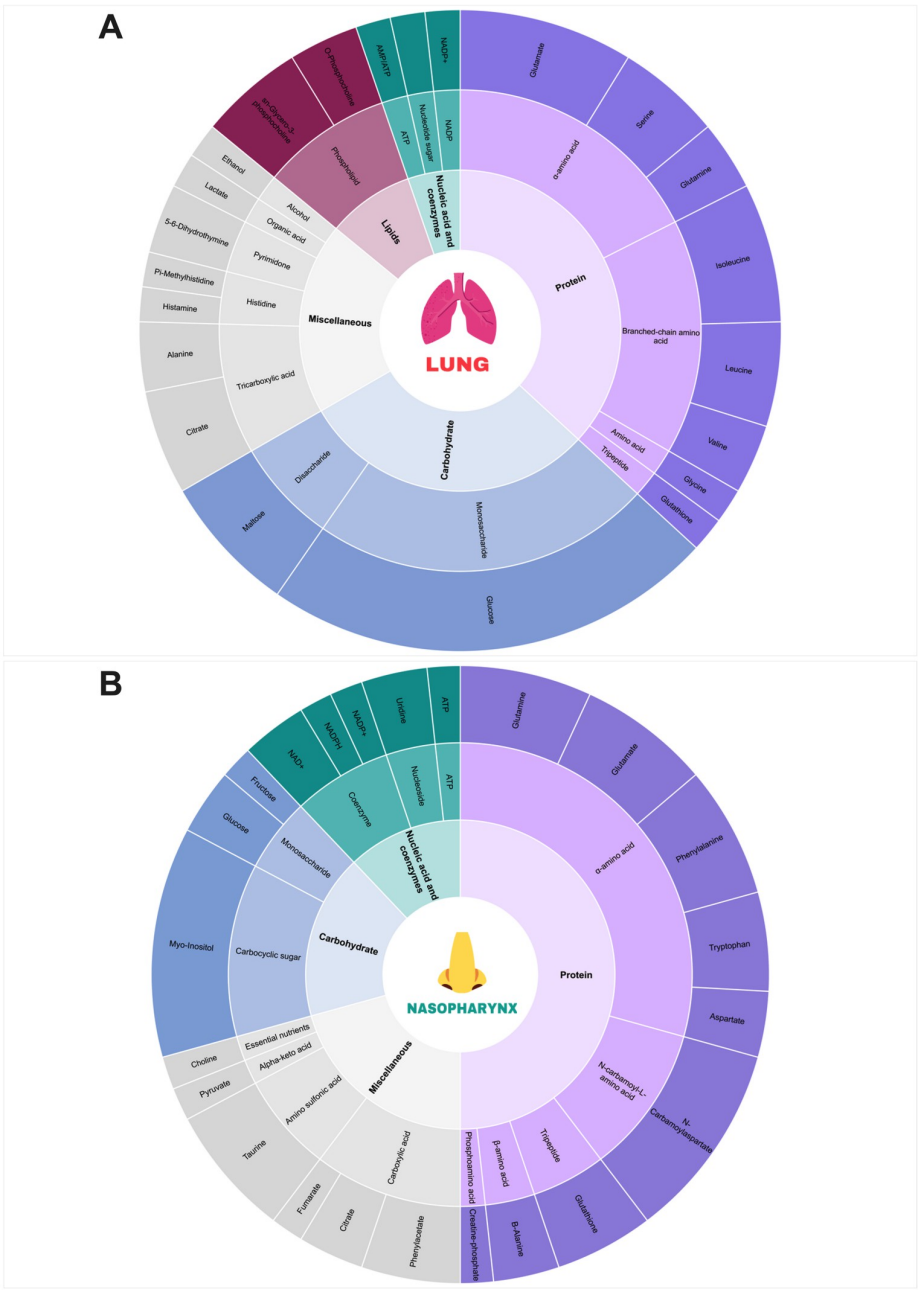
Metabolite comparison of nasopharynx and lungs from naïve mice. Sunburst plots show metabolites that were significantly enriched in **A.** lungs or **B.** nasopharynx of naïve adult Balb/c mice. The width of sub-sections of the outer ring is proportional to the number of spectral peaks associated with that metabolite that were significantly more abundant in one niche, with respect to the other. Images of the lungs and nose are taken from BioRender.com.

To gain an understanding of the metabolic landscapes first encountered by pneumococci, upon establishment of nasopharyngeal colonisation or lung infection, we first determined metabolite abundance in matched nasopharynx and lung samples from naïve, inbred Balb/c mice, using NMR (Fig 1). NMR spectra were overlaid, metabolites identified, and relative abundance compared, after normalisation of data by probabilistic quotient normalisation (Fig 1A and 1B). We identified 177 metabolite peaks from NMR spectra that showed significantly different abundance in one airway niche, as compared to the other. In the lung, there were 93 metabolite peaks with increased abundance, relative to nasopharynx (Fig 2A and Table 1). Almost half of these (46) were of unknown identity, the remaining metabolite peaks corresponding to 30 individual metabolites.

**Table 1:** Metabolites with significantly increased abundance in murine nasopharynx vs lungs. Spectral peaks (bins) that were significantly elevated in nasopharynx tissue, as compared to lungs, after data normalisation, are listed with their peak number. A full list of nasopharynx peak i.d.’s can be found in S2 Table. The total number of spectral/NMR peaks corresponding to each metabolite is also shown. Log2 fold-changes in nasopharynx abundance, relative to lung was calculated for n = 8 nasopharynx and n = 9 lung samples. P-values were corrected for multiple comparison by Bonferroni adjustment. Shading is proportional to the size of the fold change or of the p value.

Metabolite	Significant spectral bins	Total no. spectral bins	Log2 FC	Bonf adj p-value
Aspartate	314*	7	2.3	0.00779
	319		1.9	0
Tryptophan	60	5	1.0	0
	68*		0.6	0.00122
	248		0.8	0.00044
Phenylacetate	63*	6	1.0	0.00346
	64*		0.6	0.01307
	68*		0.6	0.00122
	225*		0.5	0.00006
Phenylalanine	63*	5	1.0	0.00346
	62		0.7	0.00397
	64*		0.6	0.01307
	68*		0.6	0.00122
Glutamate	359	20	0.7	0
	353*		0.6	0
	378		0.5	0
	371*		0.3	0.00203
Glutamine	343*	16	0.5	0.03419
	345*		0.5	0.01843
	371*		0.3	0.00203
	373		0.5	0.00203
N-Carbamolyaspartate	343*	10	0.5	0.03419
	345*		0.5	0.01843
	322*		1.5	0.00001
	324*		1.1	0.00012
	323		0.7	0.00003
	325		0.6	0.00029
Myo-Inositol	255*	12	1.1	0
	164		0.8	0.00011
	218		0.7	0.00008
	165*		0.6	0.00939
	216		0.6	0
	225*		0.5	0.00006
	258*		1.1	0.00006
Glucose	179	28	0.5	0.01155
	229*		0.4	0.00033
Fructose	163	1	0.9	0.02081
Histamine	255*	6	1.1	0
Creatinine	165*	2	0.6	0.00939
Taurine	258*	5	1.1	0.00006
	257		0.9	0.01654
	237		0.6	0.00006
	259		0.6	0.00001
Glutathione	191	28	0.8	0
	193*		0.6	0
	192*		0.5	0
NAD+	96	10	2.3	0
	142		0.8	0
NADP+	141	7	1.0	0.00002
Uridine	192*	6	0.5	0
	48		2.1	0.00003
	160		0.4	0.00135
UDP-glucuronate	229*	4	0.4	0.00033
N-Nitrosodimethylamine	268	2	2.0	0.00066
	193*		0.6	0
ATP	23	5	1.6	0.00001
NADPH	88	1	1.5	0.00554
Creatinine-phosphate	278	2	1.5	0.00005
Choline	264*	8	1.4	0.00011
O-Acetylcarnitine	264*	1	1.4	0.00011
Pyruvate	356	2	0.6	0.00002
Citrate	314*	9	2.3	0.00779
	324*		1.1	0.00012
Phenylacetate	63*	6	1.0	0.00346
	64*		0.6	0.01307
	68*		0.6	0.00122
	225*		0.5	0.00006
Fumarate	79	1	0.8	0.03296
Malate	353*	6	0.6	0
	322*		1.5	0.00001
Unknown	1		2.1	0.01017
	100		1.0	0.00119
	101		1.5	0
	108		2.3	0
	120		1.3	0.00003
	127		0.7	0.00002
	129		1.2	0
	130		1.0	0
	139		1.0	0.0001
	140		1.1	0
	159		0.4	0.01395
	176		0.9	0.00009
	18		2.9	0
	180		0.5	0.00036
	188		0.5	0.00001
	189		1.1	0
	2		1.5	0.01534
	20		0.7	0.04553
	226		0.6	0
	243		2.2	0.00001
	244		1.1	0.00003
	245		0.3	0.03344
	247		0.9	0
	267		2.0	0
	269		1.3	0
	270		1.8	0
	271		1.2	0
	274		0.5	0.00062
	298		0.3	0.0116
	299		0.6	0.00068
	300		0.5	0.03289
	311		0.3	0.03986
	312		1.0	0
	313		1.4	0
	317		1.6	0
	354		0.3	0.00778
	365		0.7	0.00033
	377		0.4	0.00001
	40		2.6	0.00007
	49		0.9	0.01299
	5		1.5	0.0224
	50		2.3	0
	59		0.6	0.00432
	75		3.1	0.00007
	86		1.3	0.03564
	99		1.6	0

* corresponds to shared/overlapped metabolite peaks.

In nasopharynx, we identified 84 peaks representing metabolites that were enriched, relative to their abundance in lungs (Fig 2B and Table 2), 62 of which corresponded to 36 individual metabolites.

**Table 2:** Metabolites with significantly increased abundance in murine lungs vs nasopharynx. Spectral peaks (bins) that were significantly elevated in lung tissue, as compared to nasopharynx, after data normalisation, are listed with their peak number. A full list of lung peak i.d.’s can be found in S2 Table. The total number of spectral/NMR peaks corresponding to each metabolite is also shown. Log2 fold-changes in nasopharynx abundance, relative to lung was calculated for n = 8 nasopharynx and n = 9 lung samples. P-values were corrected for multiple comparison by Bonferroni adjustment. Shading is proportional to the size of the fold change or of the p value.

Metabolite	Significant spectral bins	Total no. spectral bins	Log2 FC	Bonf adj p-value
B-alanine	332*	4	1.0	0.00163
	336*		1.3	0
Alanine	195*	13	0.3	0.0129
	401		0.5	0.00002
Glutamate	370*	20	0.7	0.00276
	196*		0.5	0.00014
Serine	169	10	0.6	0.00029
	170		0.5	0.00203
	186		0.3	0.03916
Glycine	223	2	0.4	0.0016
Aspartate	315*	7	0.6	0.00953
Glutamine	369*	16	1.0	0.00014
	370*		0.7	0.00276
	368*		0.6	0.02461
	195*		0.3	0.0129
	196*		0.5	0.00014
Isoleucine	212	8	0.7	0
	437*		0.5	0.0002
	436*		0.4	0.00021
	435		0.3	0.00197
Leucine	395	13	0.3	0.00574
	396*		0.3	0.00976
	397*		0.3	0.03197
Valine	427	6	0.5	0.00001
	428		0.5	0.00001
N-Acetylcysteine	292*	4	0.7	0.00308
N-acetylornithine	396*	2	0.3	0.00976
N-acetyllysine	397*	1	0.3	0.03197
Glutathione	331	28	1.6	0.00017
	332*		1.0	0.00163
	334		1.5	0.00046
	369*		1.0	0.00014
	330		0.9	0.00015
	287		0.8	0
	293*		0.7	0.00045
	288		0.7	0.00377
	292*		0.7	0.00308
	370*		0.7	0.00276
	285		0.7	0.00001
	339		0.7	0.00053
	368*		0.6	0.02461
	195*		0.3	0.0129
	136*		0.2	0.01178
5-6-Dihydrothymine	412	2	0.5	0.00079
	411		0.4	0.02588
Tyramine	261*	5	0.9	0.00094
Trimethylamine	293*	2	0.7	0.00045
Betaine	178*	2	0.8	0.00004
Pi-Methylhistidine	251	4	0.5	0.00333
Histamine	286	6	0.9	0
Maltose	210*	9	1.0	0.00001
	124*		1.9	0.00003
	168		0.4	0.00151
	183		0.2	0.03025
Glucose	133	28	1.9	0.00011
	132		1.4	0.00005
	230		1.3	0.00014
	232		1.1	0.00013
	182		1.0	0.00018
	233		1.0	0.00053
	181		0.9	0.00042
	261*		0.9	0.00094
	234		0.8	0.00229
	178*		0.8	0.00004
	204		0.5	0.00474
	187		0.4	0.02383
	124*		1.9	0.00003
UDP-glucoronate	115*	4	0.8	0
UDP-glucose	115*	6	0.8	0
	104*		0.9	0.03298
Lactate	406	4	0.6	0.04354
Ethanol	414	4	0.8	0.00941
Citrate	336*	9	1.3	0
	338		1.0	0.04112
	315*		0.6	0.00953
Carnitine	262*	4	1.4	0
Pantothenate	437*	4	0.5	0.0002
	436*		0.4	0.00021
O-Acetylcholine	369*	1	1.0	0.00014
O-phosphocholine	263	3	0.8	0.01777
	158		0.7	0.0001
sn-Glycero-3-phosphocholine	262*	3	1.4	0
	210*		1.0	0.00001
	153		0.9	0
GTP	136*	2	0.2	0.01178
ATP	91	5	2.7	0.04933
NADP+	152	7	0.6	0
NADH	104*	3	0.9	0.03298
Unknown	11		2.0	0.01645
	125		0.9	0.0123
	147		0.4	0.00207
	148		0.6	0.01015
	154		0.3	0.00454
	171		0.7	0
	184		0.3	0.00008
	206		0.6	0.00671
	208		0.7	0.00033
	209		1.1	0
	211		1.0	0.00001
	254		1.4	0
	294		0.7	0.00281
	296		0.6	0.01618
	303		2.5	0
	306		2.4	0
	394		0.3	0.00065
	413		0.6	0.04131
	434		0.3	0.0136
	57		1.2	0.01214
	66		0.3	0.04251

* corresponds to shared/overlapped metabolite peaks.

NMR metabolite identification in airway tissue from naïve mice highlighted broad differences between nasopharynx and lungs in the abundance of protein, carbohydrates, lipids and nucleic acids. Phospholipids, BCAA, tripeptides, and glucose were all enriched in lungs, whilst alpha amino acids, carbocyclic sugars and taurine were more abundant in nasopharynx. Pathway enrichment analysis confirmed that both BCAA biosynthesis and glutathione metabolism were more active in lungs (S1A and S2 Figs), whilst the TCA cycle, alpha amino acid pathways and arginine biosynthesis were active in nasopharynx (S1B and S3 Figs).

### Acute infection leads to rapid shifts in the lung metabolome

Next, we determined the impact of pneumococcal pneumonia on the metabolome of the murine lung. Mice were intranasally infected with a high dose, high volume *S*. *pneumoniae* D39 inoculum, leading to rapid development of pneumonia. A separate group of ten animals were infected with D39_P20-10, which had been pre-adapted to the lung environment, as part of a previous study [20]. Control mice were administered intranasal saline. We collected lung tissue at 24 hours post-infection and processed samples for NMR spectroscopy. We observed 77 metabolite peaks that were significantly differentially abundant in D39-infected mice vs saline controls, of which 33 peaks (corresponding to 18 metabolites) were less abundant in infection and 44 peaks (corresponding to 17 metabolites) were more abundant (Table 3).

**Table 3:** Metabolite abundance changes in lung during pneumococcal pneumonia. Metabolite peak numbers are listed. Fold-changes are based on n = 9 D39-infected lung samples, n = 8 D39_P20-10-infected lung samples, and n = 9 PBS-treated lung samples. P-values were corrected for multiple comparison by Bonferroni adjustment.

		PBS vs D39		PBS vs D39_P20-10		D39 vs D39_P20-10	
Peak No.	Metabolite	Log2 FC	P value	Log2 FC	P value	Log2 FC	P value
111	Unknown	1.85	0.00001	1.76	0.00003		
63*	Phenylacetate/Phenylalanine	1.43	0.00007	1.31	0.00051		
51	Unknown	1.15	0.00586	1.00	0.02946		
50	Unknown	1.13	0.00025	0.73	0.04161		
64*	Phenylacetate/Phenylalanine/5-Hydroxyindole-3-acetate	1.12	0.00031	1.09	0.00065		
67*	Phenylacetate/Phenylalanine	1.06	0.00048	1.08	0.00050		
120	Unknown	1.01	0.00128	0.99	0.00212		
68*	Phenylacetate/Phenylalanine/ Tryptophan	0.74	0.00181	0.86	0.00032		
269	Unknown	0.66	0.00038	0.63	0.00108		
441	Unknown	0.66	0.00364			-0.45	0.03687
75	Unknown	0.60	0.00501	0.43	0.07546		
268	B-Alanine	0.58	0.00082	0.59	0.00097		
100	Unknown	0.58	0.00792	0.54	0.01807		
72	Tyrosine	0.58	0.01681	0.69	0.00426		
274	Unknown	0.54	0.00542	0.55	0.00551		
300	Unknown	0.54	0.00169	0.44	0.01397		
176	Unknown	0.49	0.00826				
366	Glutathione	0.48	0.00018	0.37	0.00477		
299	Unknown	0.45	0.00391	0.40	0.01404		
398	Unknown	0.45	0.00124	0.42	0.00329		
267	Unknown	0.40	0.00844	0.45	0.00375		
168*	Maltose/Unknown	0.36	0.00001	0.28	0.00063		
167*	Serine/Nicotinurate	0.36	0.00019	0.28	0.00352		
185*	Glucose/Maltose	0.33	0.00484	0.32	0.00801		
191*	Glutathione/Unknown	0.30	0.01537	0.40	0.00142		
175	Creatine	0.30	0.00257	0.25	0.01405		
169	Serine	0.29	0.00027	0.21	0.00920		
325*	N-Carbamoylaspartate/Unknown	0.28	0.01405	0.29	0.01487		
146*	ATP/NADP+	0.26	0.01401	0.28	0.00970		
393	Unknown	0.22	0.04141	0.32	0.00337		
172*	Creatine-phosphate/Serine/Glycylproline	0.16	0.00106	0.14	0.00649		
173	Glycolate	0.13	0.00558	0.10	0.04248		
174*	Tyrosine/Serine/Unknown	0.13	0.00628	0.11	0.02243		
216	Myo-inositol	-0.11	0.00605	-0.11	0.00964		
372*	Glutamine/Unknown	-0.14	0.00375	-0.10	0.04741		
187*	Glucose/Unknown	-0.29	0.01012	-0.29	0.01328		
245	Unknown	-0.30	0.00376				
215	Unknown	-0.30	0.00010	-0.16	0.03076		
213*	Unknown/Ethanol/Maltose	-0.31	0.00002	-0.17	0.00916	0.14	0.04820
225*	Myo-inositol/Phenylacetate	-0.32	0.00053	-0.37	0.00019		
179*	Unknown/Glucose	-0.34	0.00871	-0.37	0.00587		
186	Serine	-0.34	0.00090	-0.37	0.00056		
97	Unknown	-0.35	0.04236	-0.51	0.00597		
162	Lactate	-0.38	0.00077	-0.35	0.00207		
222*	Maltose/Glycylproline	-0.40	0.00109				
406	Lactate	-0.40	0.01596	-0.46	0.00778		
204*	Glucose/Unknown	-0.41	0.00071	-0.45	0.00041		
182	Glucose	-0.43	0.00030	-0.45	0.00024		
260*	cis-Aconitate/Glucose/Tyramine	-0.44	0.00171	-0.38	0.00618		
292*	Glutathione/N-N-Dimethylglycine/N-Acetylcysteine	-0.48	0.00190	-0.41	0.00758		
254	Unknown	-0.51	0.00248	-0.44	0.00876		
200	Glutamate	-0.53	0.00003	-0.44	0.00029		
178*	Unknown/Glucose/Betaine	-0.54	0.00003	-0.53	0.00005		
198*	Gutamine/Glutamate	-0.56	0.00045	-0.45	0.00366		
207*	Pi-Methylhistidine/Unknown	-0.57	0.00004	-0.56	0.00007		
181	Glucose	-0.60	0.00002	-0.60	0.00003		
206	Unknown	-0.62	0.00002	-0.60	0.00005		
231*	Glucose/Unknown	-0.62	0.00542				
234	Glucose	-0.62	0.00005	-0.61	0.00008		
80	Unknown	-0.68	0.00094	-0.46	0.01893		
261*	Tyramine/Glucose	-0.69	0.00002	-0.69	0.00003		
224	Myo-inositol	-0.69	0.00000	-0.54	0.00001		
124*	Maltose/Glucose	-0.71	0.00002	-0.73	0.00003		
133	Glucose	-0.72	0.00002	-0.73	0.00002		
132	Glucose	-0.72	0.00003	-0.75	0.00003		
233	Glucose	-0.73	0.00001	-0.70	0.00002		
230	Glucose	-0.73	0.00002	-0.75	0.00003		
232	Glucose	-0.74	0.00002	-0.75	0.00002		
240	Glucose	-0.77	0.00008	-0.61	0.00085		
316	Unknown	-0.79	0.00180	-0.56	0.01958		
5	Unknown	-0.81	0.00864	-0.60	0.04563		
338	Citrate	-0.83	0.00203	-0.58	0.02181		
238*	Unknown/Glucose	-0.92	0.00243	-0.86	0.00475		
7	NAD+	-0.96	0.00447	-0.66	0.03909		
57	Unknown	-1.10	0.00000	-0.77	0.00002		
227*	Unknown/Pantothenate	-1.30	0.00000	-1.00	0.00005		
239	Glucose	-1.81	0.00696				

* corresponds to shared/overlapped metabolite peaks.

**Fig 3:**
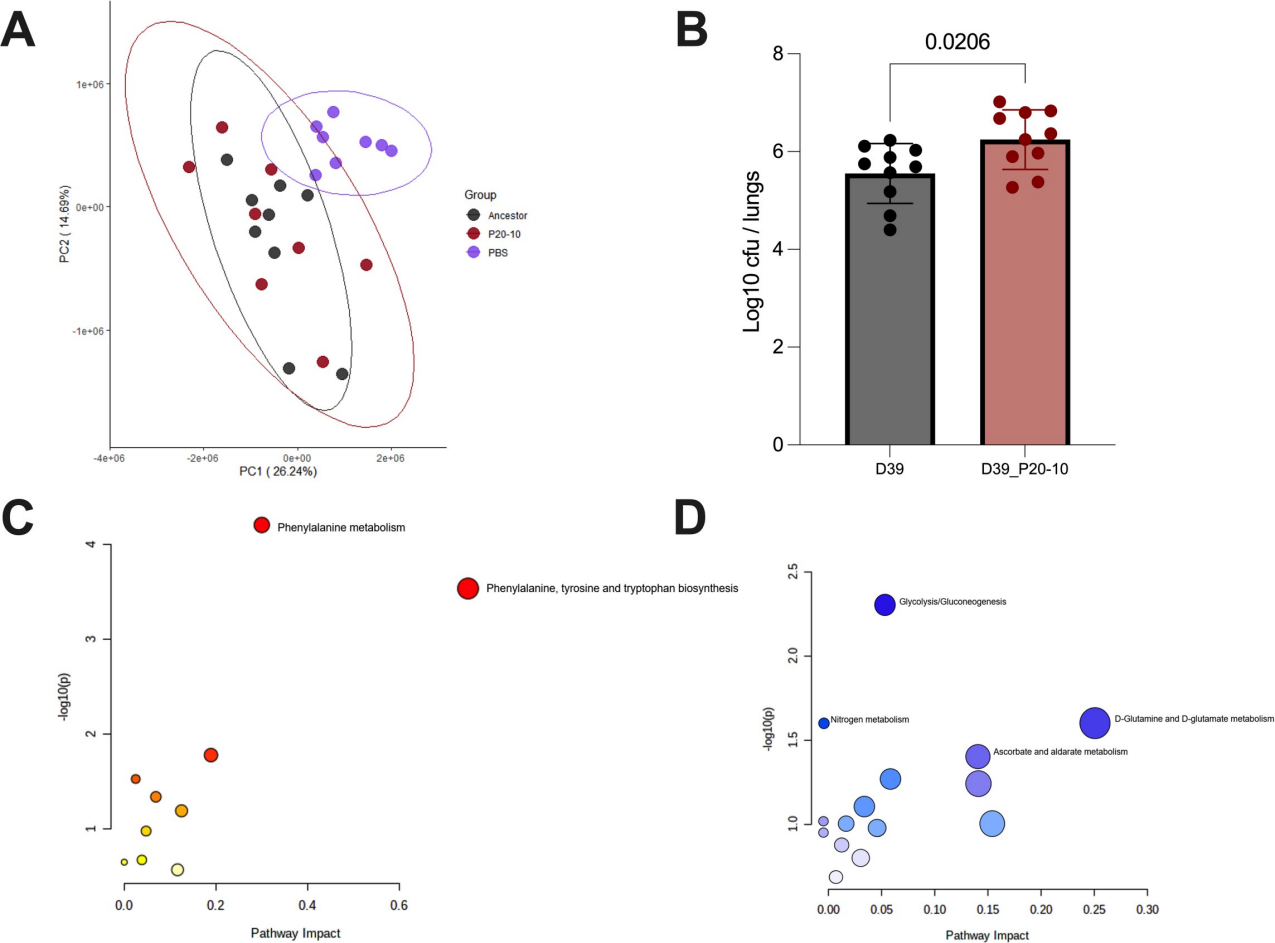
The effect of acute *Streptococcus pneumoniae* lung infection on the lung metabolome. **A.** Principal component analysis of lung NMR metabolomics data from sham-infected mice (PBS, purple), those infected with the D39 ancestor (Ancestor, grey) and those infected with a lung-adapted D39 (P20-10, red). Mice were infected with 50 μl PBS (sham group) or 50 μl PBS containing 1 x 10^6^ colony forming units *S*. *pneumoniae*. Lungs were extracted at 24 hours post-infection. N = 9 for the PBS group and N = 8 for both D39 and P20-10. **B.** Lung infection burden at 24 hours post-infection. Data show log10 colony forming units (CFU). Each data point represents an individual animal. P value is from unpaired T test. **C.** Pathway enrichment analysis, showing those metabolic pathways that are upregulated in lungs during infection. **D.** Pathways that are downregulated in lungs during infection.

Of the 77 identified peaks, 71 were also significantly different in comparisons of D39_P20-10 vs saline controls (Table 3). Only two peaks differed significantly in comparisons of D39 vs D39_P20-10 (Table 3). Neither of these peaks have definitive metabolite identities assigned, although one, which was moderately enriched during D39 infection vs D39_ P20-10 infection, is in a region of spectral overlap annotated as maltose, ethanol and an unidentified metabolite. Thus, whilst infection had a substantial impact on the lung metabolic landscape, there was little difference between infection with D39 and the closely related strain D39_P20-10, despite the enhanced lung colonisation potential of the latter (Fig 3A and 3B). Phenylalanine metabolism was upregulated in the lung during infection with both strains (Fig 3C), whilst metabolites associated with glycolysis and gluconeogenesis, principally glucose, and those associated with glutamine and glutamate metabolism, were found in reduced abundance (Fig 3D).

### Upper airway colonisation only minimally perturbs the nasopharyngeal metabolome

**Fig 4:**
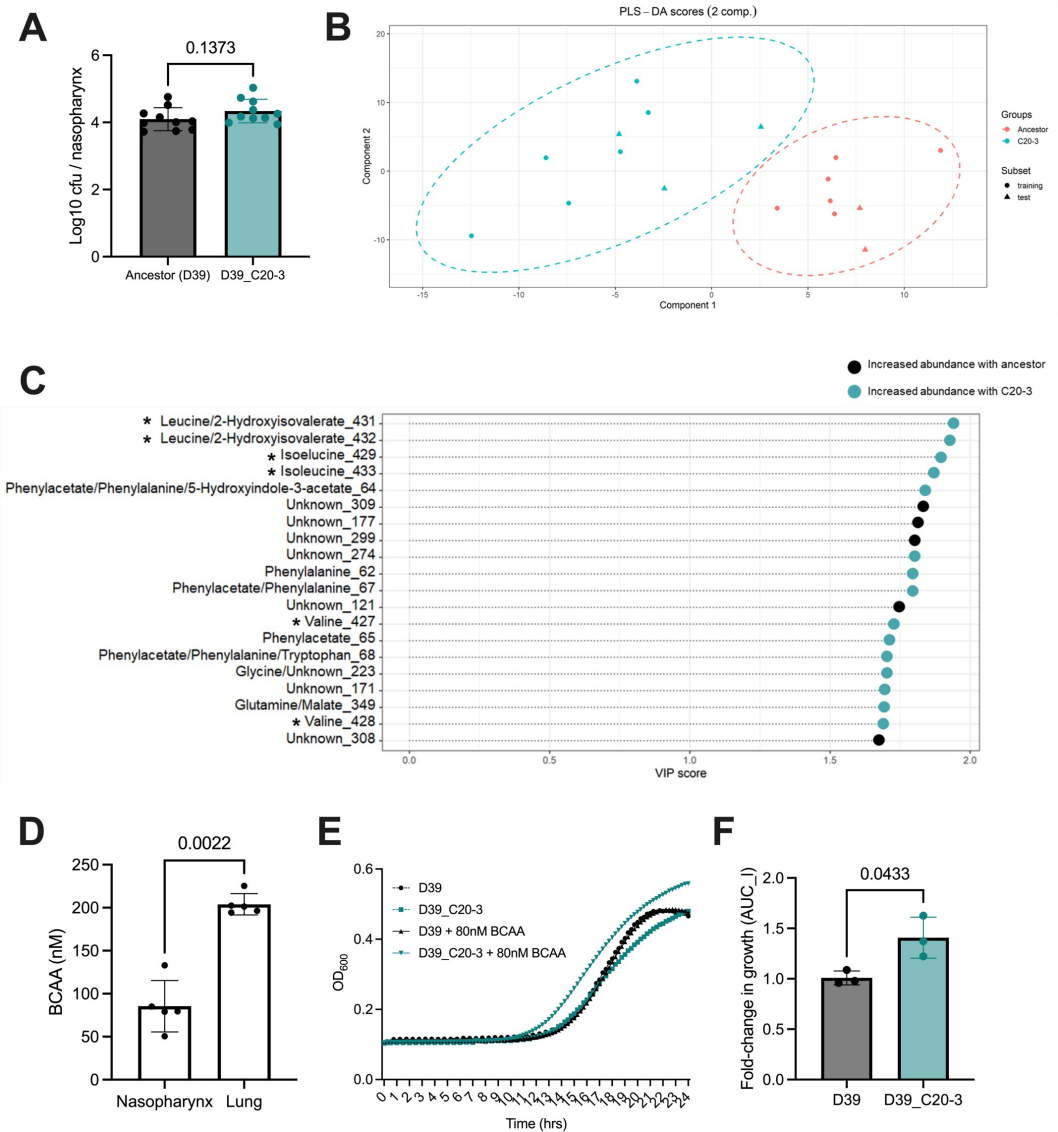
Partial-least squares discriminant analysis of the nasopharyngeal metabolome during upper airway carriage with *Streptococcus pneumoniae*. **A.** Nasopharynx infection burden at 7 days post-infection in mice infected with D39 or nasopharynx-adapted D39-derivative C20-3. Mice were infected with 1 x 10^5^ colony forming units of *Streptococcus pneumoniae* in 10 μl PBS. Nasopharynx was excised, post-mortem, at 7 days post-infection. Data show log10 colony forming units in the nasopharynx. Each data point represents an individual animal and p value is from unpaired t test analysis. **B.** Multivariate partial least squares discriminant analysis (PLS-DA) was carried out to determine predictive models that distinguish metabolite profiles of nasopharynx tissue infected with D39 (ancestor) (red) vs infection with a nasopharynx-adapted D39 (C20-3) (teal). In this scores plot, the data have been represented by two PLS-DA components, the model is fit to a random subset of data and evaluated on the remaining (circles for training and triangles for test). The test examples are inside the clusters of training examples, indicating a good model fit. **C.** The top 20 ranked VIP scores for NMR metabolite spectra peaks used by the PLS-DA model to distinguish between nasopharyngeal samples from mice infected with the ancestor (D39) and those infected with nasopharynx-adapted derivative C20-3. The length of the dashed line represents the relative value that each metabolite is given within the model. The colour of the circle indicates whether each metabolite was more abundant in nasopharynx during infection with D39 (black) or C20-3 (teal). Asterisks (*) denote peaks associated with branched-chain amino acids. **D.** Branched chain amino acid concentration, determined in nasopharynx and lungs of five naïve mice, using a colourimetric assay. P-value determined by two-tailed T-test. **E.** Growth of D39 and D39_C20-3 in 20% M17 media or in 20% M17 adjusted to 80 nM BCAA, to reflect nasopharynx concentrations. **F.** Fold-change in growth rate of D39 and D39_C20-3 in 20% M17 induced by adjustment of BCAA to 80 nM. Growth rate was determined by calulcating the area under the logistic curve from data in **E.** Three biological replicates were performed. P value is from two-tailed T test.

The shifts in metabolite profiles during pneumonia likely result, at least in part, from the induction of host inflammatory and innate immune responses. We next sought to determine whether colonisation of nasopharynx ‐ which is not associated with acute inflammation–would similarly perturb the local metabolome. Mice were intranasally infected with a low dose, low volume inoculum of D39, or D39_C20-3, a strain that had been pre-adapted to the nasopharynx environment in a previous study [20]. Control animals were given saline intranasally. We allowed colonisation to establish over 7 days, before collecting nasopharynx for NMR analysis. Although D39_C20-3 has higher long-term colonisation potential in nasopharynx than D39, at 7 days post-infection, nasopharyngeal bacterial numbers are comparable between the two groups of infected mice (Fig 4A). In clear contrast to lower airway infection, colonisation of the nasopharynx was not associated with obvious changes in metabolic profiles (S4A Fig). Only two metabolite peaks were significantly different in ANOVA comparison of the three groups. The two peaks, representing tryptophan and isoleucine, were elevated in the nasopharynx of mice infected with D39_C20-3, as compared to uninfected mice (p < 0.0001 for both peaks) and also as compared to those infected with D39 (p = 0.0012 for tryptophan, p = 0.0065 for isoleucine) (S4B and S4C Fig). Thus, colonisation only minimally perturbed the nasopharyngeal niche, with change in the metabolic landscape only detectable during infection with a strain of high colonisation potential. The observation that the magnitude of the metabolic change in the nasopharynx during infection was strain-dependent suggested it might be possible to distinguish between infections caused by pneumococci of differing colonisation potentials, through analysis of the nasopharyngeal metabolome.

### Nasopharyngeal carriage with a pneumococcal strain of high colonisation potential is associated with signatures of branched chain amino acid metabolism

Using the metabolomics data derived from D39-infected and D39_C20-3-infected nasopharynx, we implemented a partial least squares discriminant analysis (PLS-DA) approach to distinguish the respective NMR spectra. PLS-DA is a multivariate dimensionality reduction approach that is analogous to a supervised principal component analysis, but where the class labels of input data are known [21]. It can thus be useful to deconvolute ‘noisy’ data, such as those obtained from NMR.

The resulting PLS-DA model was able to successfully distinguish between NMR spectra derived from D39-infected nasopharynx and those derived from D39_C20-3-infected tissue (Fig 4B). The model was built with 2 components and the cross validation AUROC score was 1 (S1 Dataset). The VIP scores indicate that the model relied heavily upon spectral peaks associated with BCAA to distinguish between traces. The four peaks with the heaviest weighting in the model were all associated with leucine and isoleucine and two peaks associated with valine were also amongst the top 20 peaks used in the model. All six of these peaks were more abundant in D39_C20-3-infected nasopharynx than in D39-infected nasopharynx (Fig 4C). Five of the remaining top 20 peaks were associated with phenylalanine or phenylacetate, and these too were more abundant in D39_C20-3-infected tissue (Fig 4C).

The PLS-DA model (Fig 4), together with the NMR data from naïve nasopharynx and lungs Fig 2) suggested that whilst BCAA were rare in nasopharynx, their relative abundance might influence pneumococcal carriage. We quantified BCAA levels in nasopharynx and lungs of naïve mice, using a colourimetric detection kit (Fig 4D). BCAA were more than 2-fold more abundant in lung, as compared to nasopharynx (mean 204 nmol vs 81 nmol/tissue). When D39 and D39_C20-3 were grown under growth-limiting conditions, with BCAA at 80 nM, D39_C20-3 showed evidence of a fitness advantage (Fig 4E and 4F), consistent with its *in vivo* phenotype.

PLS-DA analysis for the lung datasets failed to deliver a model than could reproducibly distinguish between D39-infected lung and P20-10-infected lung (AUROC <0.8) but PBS control lung samples could be distinguished from both infection groups (AUROC = 1 for both comparisons). Model discrimination of infected from uninfected lung relied on metabolite peaks including those identified as taurine, glucose and myo-inositol (S2 Dataset).

### High-colonisation potential is associated with increased branched chain amino acid acquisition or biosynthesis in D39

**Fig 5:**
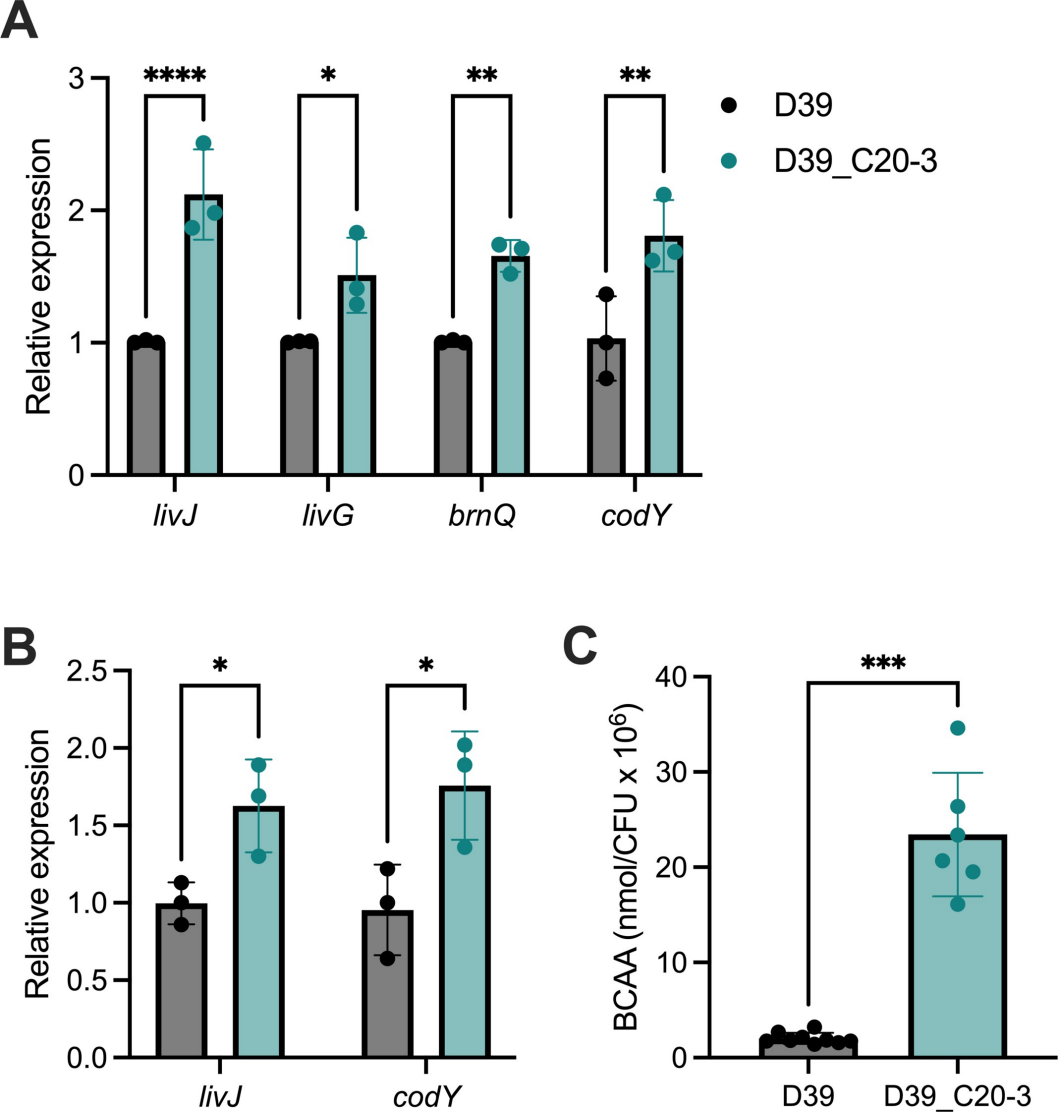
Altered branched chain amino acid metabolism in nasopharynx-adapted pneumococci. Expression of genes associated with branched chain amino acid synthesis, transport, or sensing, in nasopharynx-adapted D39 lineage D39_C20-3, relative to the D39 ancestor from which it was derived, in (**A**) nutrient broth and (**B**) excised mouse nasopharynx from 7 days post-infection. Expression levels were determined by qRT-PCR, using the 2^-ΔΔCt^ method. *ddl* was used as a housekeeping gene for normalisation of expression. Data analysis is by two-way ANOVA with Sidak’s multiple comparison test vs D39. n = 3 biological replicates per gene, per strain. Each biological replicate presented is the mean of 2–3 technical replicates (**A**) or represents data from a single animal (**B**). **C.** Branched chain amino acid (BCAA) abundance in mid-log bacterial cultures. BCAA abundance was determined from pelleted and lysed bacteria by colourimetric assay and normalised to culture density (colony forming units–CFU). Data are from 9 (D39) or 6 (D39_C20_3) independent cultures. Data analysis is by unpaired t-test. * = p<0.05, ** = p<0.01, *** = p<0.001, **** = p<0.0001.

Given the vast excess of host material in excised nasopharynx, as compared to that derived from pneumococci, our expectation was that metabolites detected by NMR would be predominantly host-derived. The effects of infection on the metabolic landscape were presumed to be the result of the host response to pneumococci. However, BCAA were scarce in nasopharynx samples derived from naïve animals (Fig 2) and so the possibility remained that their elevation during D39_C20-3 infection might be a signal of amino acid biosynthesis in the bacteria or else result from the liberation of host materials by microbial-driven processes. We measured the expression of BCAA transport genes in D39 and D39_C20-3, during growth in broth. Genes of the *liv* operon, encoding a branched chain amino acid ABC transport system [22], were upregulated in D39_C20-3, relative to D39, as was another BCAA transporter gene, *brnQ* (Fig 5A). Control of *brnQ* is thought to be mediated, at least in part, by CodY, due to the presence of a CodY-binding site upstream of the *brnQ* transcriptional start site [23]. CodY has been demonstrated to be BCAA-inducible in a number of Gram-positive species [24] and, here, its expression was found to be elevated in D39_C20-3, relative to D39, (Fig 5A). In accordance with these *in vitro* data, expression of both *livJ* and *codY* was found to be elevated in D39_C20-3, relative to D39, when qRT-PCR was performed with RNA extracted from mouse nasopharynx, at 7 days post-infection (Fig 5B).

Analysis of genome sequencing data for D39_C20-3 [20] (S1 Table) did not reveal an obvious driver of these gene expression changes, but it appears to be a common signature of adaptation to nasopharynx, at least in D39-derived experimental evolution lineages. We measured expression of the same genes in three other high nasopharyngeal colonisation potential D39-derived strains, from the same previous study [20], and each of these showed significant upregulation of at least one of the four genes investigated (S5A Fig).

To assess the combined effect of BCAA acquisition and biosynthesis, we quantified BCAA levels in pneumococci grown in broth. D39_C20-3 accumulated significantly higher quantities of BCAA than D39 during mid-exponential growth (Fig 5C), as did all three of the other high colonisation potential D39-derived strains tested (S5B Fig). Thus, increased BCAA accumulation in pneumococci is a signature of high-colonisation potential, in the D39-derived lineages tested here.

Five of the top 20 peaks used by the PLS-DA model to distinguish D39 from D39_C20-3 infection represented phenylalanine. To determine whether, like BCAA, phenylalanine elevation during D39_C20-3 nasopharyngeal carriage might be the result of increased synthesis by the pneumococci, we quantified phenylalanine levels in mid-log phase cultures of D39, D39_C20-3 and three other nasopharynx-adapted lineages (S5C Fig). Phenylalanine abundance was comparable in D39 and D39_C20-3 cultures, suggesting its elevation during nasopharyngeal carriage in D39_C20-3 infected mice may be due to host-derived processes. All strains tested showed an enhanced duration of stable nasopharyngeal carriage, relative to the D39 ancestor (S5D Fig).

Collectively, these data highlight the compartmentalisation of the metabolic landscape of the respiratory tract and demonstrate the utility of NMR approaches for exploring infection-driven changes in metabolite bioavailability. Furthermore, our findings add to the growing evidence that BCAA signalling and metabolic pathways are key contributors to microbial fitness within the airway environment [11,25–28].

## Discussion

**Fig 6:**
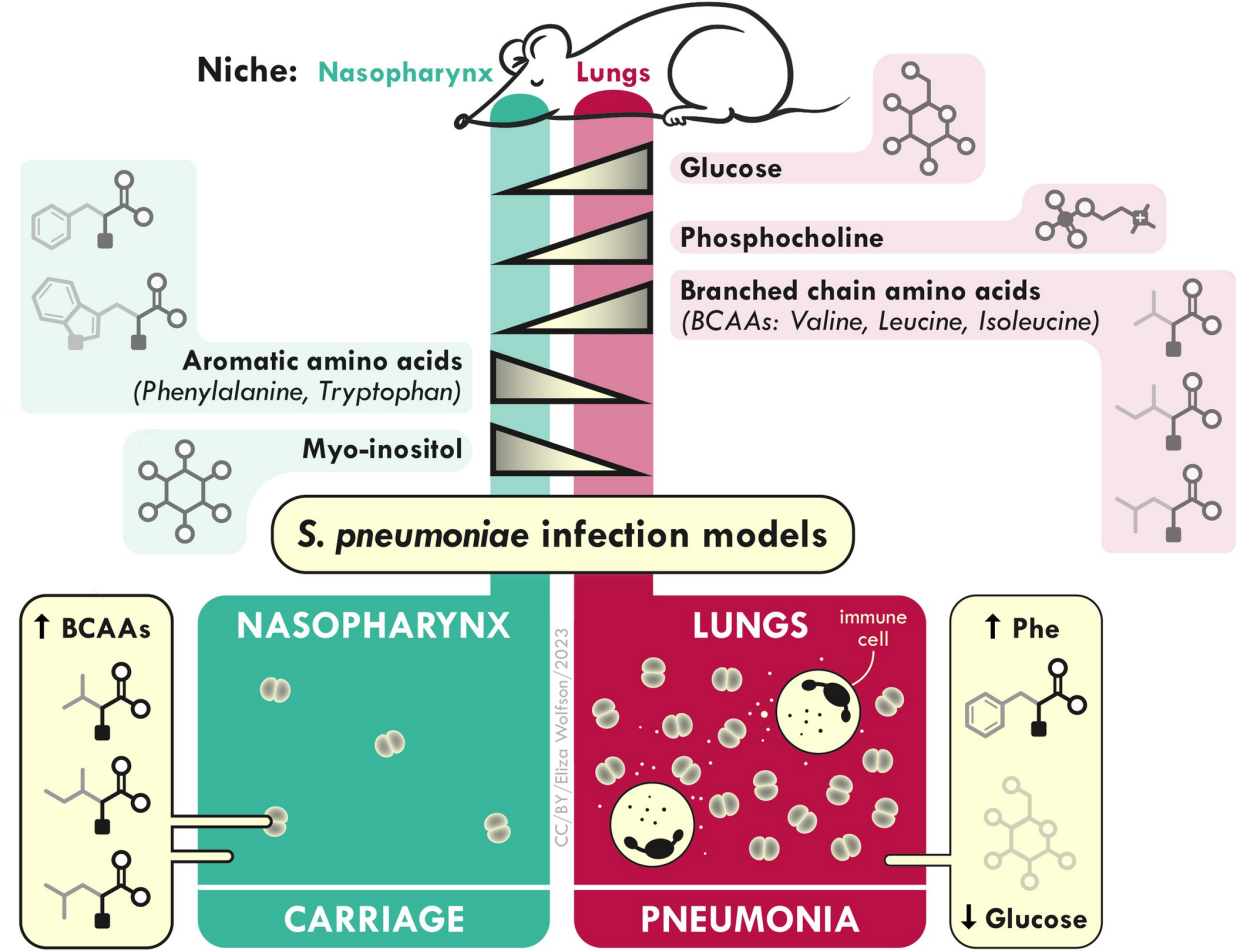
Nasopharynx and lung metabolic profiles in health and during pneumococcal infection. Gradients of key metabolites in the airways of naïve mice are depicted. In the bottom half of the figure, the effects of asymptomatic nasopharyngeal carriage and of acute lung infection (pneumonia) are shown. Carriage is associated with a local elevation of branched-chain amino acids (BCAA) that is at least partially driven by bacterial BCAA synthesis. Pneumonia stimulates elevations in lung phenylalanine and depletion of lung glucose. Both effects may be a consequence of innate immune and inflammatory responses in the lung environment. This image was generated by Dr Eliza Wolfson (https://lizawolfson.co.uk).

Here, we sought to characterise differences in the metabolic landscapes of two airway environments that can be colonised by the major human pathogen *Streptococcus pneumoniae*. Our findings suggest that, whilst a broad suite of metabolites are shared between nasopharynx and lungs, their relative abundance in each niche can differ considerably. Furthermore, pneumococcal infection alters the local metabolic environment in a manner proportional to the density of infection and the magnitude of the host response (Fig 6). Nasopharyngeal carriage, associated with low bacterial numbers and a muted host inflammatory response, caused only minimal disruption to the upper airway metabolic profile, while acute lung infection, with a high infection burden and induction of robust innate immune responses, resulted in considerable metabolic perturbation.

We opted to conduct these studies in mice, to enable us to collect matched lung and nasopharynx from individual animals, and to afford us the experimental flexibility to separately assess the impact of pneumococcal carriage and pneumonia. Some of the findings reported here may not translate to human infection, but there is much similarity in the physiology and central metabolism of mouse and man and previous NMR studies have reported metabolic similarities in murine and human airway samples [29]. Indeed, several of the findings here have parallels in clinical studies. Phenylalanine was found at increased abundance in lung during pneumococcal pneumonia and was also one of the metabolites used to discriminate upper airway carriage with the nasopharynx-adapted D39_C20-3 strain, relative to that with the D39 ancestor, being more abundant in the former. Increased phenylalanine levels associated with pneumococcal infection have been documented in both pneumonia [30,31] and sepsis [32], and have been ascribed to increased skeletal muscle catabolism [32]. Similarly, the dramatic drop in airway glucose observed during pneumococcal lung infection in mice mirrors observations of hypoglycaemia in patients with pneumococcal pneumonia [33]. The elevated airway glucose concentrations associated with chronic obstructive pulmonary disorder (COPD), both at baseline and during exacerbation, are thought to contribute to infection susceptibility and COPD airway samples with high glucose support increased *Pseudomonas aeruginosa* growth *in vitro* [34]. Glucose depletion within the airways has been suggested to be an innate immune defence aimed at restricting the growth of bacterial pathogens [35].

To increase the quantity of material available for analysis, and to ensure reproducibility of sample collection, our studies were performed with nasopharynx and lung tissue, rather than with nasal washes and bronchoalveolar lavage. Identified metabolites will therefore have included both intracellular and extracellular material. Much of the intracellular material may not be bioavailable to pneumococci during infection, other than those metabolites released during bacterially-driven or inflammatory-mediated tissue damage. Future work focussing on the extracellular compartment would add to the data presented here, as would targeted studies using a high sensitivity mass spectrometry approach.

The centrality of BCAA to bacterial colonisation of the human host is reflected in the sensitivity of bacterial central metabolic regulators to subtle changes in BCAA bioavailability [12]. Those same central regulators also control multiple aspects of virulence [36]. Therefore, both the inherent differences in BCAA abundance between nasopharynx and lungs that we observed, and the changes in abundance induced by infection, have potential to impact infection outcomes. The transcriptional regulator CodY is conserved amongst Gram-positive firmicutes, and is activated directly by BCAA in pneumococcus [13] and by BCAA and GTP in other species [37]. CodY represses the expression of a broad regulon but has also been demonstrated to activate genes in some species [38]. Broadly, it is thought to negatively regulate virulence in Gram-positives, but findings in pneumococcus have been mixed, with a *codY* mutant showing reduced colonisation but no defect during systemic infection [13]. What is clear, however, is that the CodY regulon is controlled in a hierarchical fashion, such that subtle changes in BCAA concentration may affect the expression of some genes but not others [39]. Therefore, changes in BCAA concentration as pneumococci move from upper to lower airways, or as a consequence of host responses to infection, might influence virulence and, therefore, infection outcomes. We detected increased abundance of BCAA in lungs, relative to nasopharynx, but also elevations in nasopharynx during infection with a D39 strain that had been pre-adapted to the nasopharyngeal environment and that shows an increased duration of carriage, relative to the ancestral D39 [20]. A PLS-DA model, developed to distinguish NMR traces from nasopharynx of mice infected with D39 from those infected with the high colonisation potential strain, relied heavily on BCAA for discrimination. Leucine, isoleucine and valine were all elevated during infection with the nasopharynx-adapted strain. Thus, BCAA abundance in nasopharynx appears to be positively correlated with nasopharyngeal colonisation potential, at least in these two closely related strains that differ only by 11 fixed mutations. Further studies are needed to determine whether this observation will be more broadly applicable across pneumococci, including clinical isolates, and whether it holds true in human infection. The experimental human pneumococcal challenge model provides opportunity to test whether upper airway BCAA abundance during early colonisation is predictive of subsequent carriage duration [40].

Further study is also necessary to explore how BCAA metabolism might be co-regulated with carbohydrate metabolism, which also plays a prominent role in nasopharyngeal colonisation [41]. Pneumococcal carbon-catabolite repression (CCR) is alleviated during nasopharyngeal carriage, via down-regulation of the catabolite control protein ccpA [42]. However, ccpA has a broad regulon and Δ*ccpA* mutants show an attenuated ability to establish colonisation and to cause disease [42,43]. Whether the ccpA regulon includes genes involved in BCAA metabolism is not clear, but phenotypes associated with successful colonisation, including reduced capsule expression and increased adhesion potential are observed both during CCR de-repression [42] and in the nasopharynx-adapted lineages used in this study [20].

Although host material was likely several orders of magnitude more abundant than pathogen-derived metabolites in samples used for NMR, there remained the possibility that microbial metabolism might directly contribute to the observed differences between naïve and infected animals and between infections with different pneumococcal lineages. The heavy weighting given to BCAA peaks in the PLS-DA model used to distinguish D39-infected nasopharynx from D39_C20-3 infected tissue prompted us to quantify BCAA in cultures of these strains and to assess expression of *codY* and BCAA transport genes. BCAA were more abundant in mid-log cultures of the nasopharynx-adapted strain, relative to the ancestor and, accordingly, *codY* was also being expressed at a higher level. We observed elevated expression of BCAA transport genes *brnQ* and genes of the *liv* operon in D39_C20-3. These genes are subject to negative regulation by *codY*, enabling reduction in BCAA uptake in environments rich in those resources [13,22,24]. Their upregulation, alongside that of *codY*, was therefore somewhat surprising and may reflect the sensitive and hierarchical control of gene expression by CodY. Similar patterns of BCAA abundance and gene expression in other D39-derived, nasopharynx-adapted lineages suggests that these changes may be a generalised adaptation to nasopharyngeal carriage, although no obvious genetic drivers of these changes are apparent from genome sequence analysis [20].

Whilst PLS-DA analysis enabled discrimination between D39- and C20-3-infected nasopharynx, a comparable approach taken with the lung datasets failed to yield a model that could distinguish between D39 and P20-10 infection, despite the enhanced lung-infection potential of the latter. The effects of infection on the metabolic landscape of the lung were more pronounced than those observed in nasopharynx, likely as a result of the robust inflammatory responses associated with acute infection. This might have masked strain-specific effects. The short time-frame of the acute infection model may also not be sufficient for those differences to manifest.

Collectively, these data highlight the metabolic challenges faced by pneumococci as they move between niches within the host. The centrality of BCAA to bacterial metabolism and virulence, their differential abundance in upper and lower airway niches, and the ability of high-colonisation potential pneumococci to enrich the BCAA pool in nasopharynx suggest that these amino acids might be key metabolic determinants of virulence and infection outcomes.

## Methods

### Ethics statement

All animal infections were performed at the University of Liverpool, with prior approval from the UK Home Office (project licence PP2072053) and the local animal welfare ethical review board (AWERB). The principles of the Declaration of Helsinki were observed throughout. Mice were housed in individually ventilated cages, with access to food and water *ad libitum*. Environmental enrichment was provided in all cages and mice were acclimatised to the animal unit for at least 7 days before use. Mice were randomly allocated to cages on arrival in the animal unit by staff with no role in study design. For experiments reported in this manuscript, individual mice were considered as the experimental unit. Sample sizes, controls and statistical analyses are detailed in the figures and accompanying legends. Any samples excluded from analyses following quality control testing are detailed in the manuscript text.

### Bacteria

All *S*. *pneumoniae* used in this study were derivatives of the serotype 2 strain D39. An in-house D39 (D39N) [44] was used, alongside derivatives of that strain that had been pre-adapted to mouse nasopharynx and lungs, in a previous study, via serial passage through infection models [20]. The nasopharynx adapted strains used were D39_C20-1, D39_C20-3, D39_C20-6 and D39_C20-10. The lung adapted strain used was D39_P20-10.

### Mouse infections

For NMR profiling of lung tissue there were three groups each containing ten mice: 1. A lung control group. 2. A lung test group that was infected with D39 wild type *S*. *pneumoniae*. 3. A lung test group that was infected with a D39-derived, lung adapted lineage (D39_P20-10). To induce pneumonia, Balb/c inbred mice (4–6 weeks old females) (Charles River, Oxford, UK) were infected intranasally under light anaesthesia, using a mix of oxygen and isoflurane, with 1 × 10^6^ colony forming units (CFU), in 50 μl PBS. For the control group, mice were administered 50 μl of PBS intranasally. Lung tissue samples were collected 24 hours post infection.

For NMR profiling of nasopharynx tissue there were three groups, each containing ten mice: 1. A nasopharynx control group. 2. Nasopharynx test group infected with 10 μl D39. 3. A nasopharynx test group infected with 10 μl of a D39-derived, nasopharynx adapted lineage (D39_C20-3). To induce nasopharyngeal carriage, mice were infected intranasally under light anaesthesia, using a mix of oxygen and isoflurane 1 x 10^5^ CFU in 10 μl PBS. For the control group, mice were administered 10 μl of PBS intranasally. Nasopharynx tissue samples were collected seven days post infection. In both the lung infection and nasopharyngeal carriage models, separate groups of mice were used for confirmation of infectious burden. These were treated as above, but dissected tissue was homogenised with an IKA handheld tissue homogeniser and then serially diluted in PBS and plated on blood agar, to determine pneumococcal density within tissues.

### Tissue dissection

In order to interleave batches of different sample groups, lungs were harvested from five mice at a time in the following order, five mice from the control group, and then five mice from each test condition until all mice were culled and lungs harvested. The intact lung samples were dipped in ice-cold PBS solution, pH 7.4, to remove any blood surrounding the organ. Lungs were placed into 1.5 ml bead beating tubes, snap frozen in liquid nitrogen and stored at -80°C until metabolite extraction (for a period of no more than 3 months). From the lung control group, the nasopharynx tissues were also dissected, as soon as the lungs were removed, in order to compare the NMR profiles of the lung with the nasopharynx, in the absence of pneumococcal infection.

For the nasopharynx groups, mice were culled and nasopharynx tissue was harvested from two mice at a time in the following order, two mice from the control group, and then two mice from each test condition until all mice were culled and tissue samples collected. Nasopharynx dissection was carried out following the revised guides for organ sampling and trimming in rats and mice [45]. Tissue was collected in a region between the posterior part of the upper incisors and the second palatine crest. All tissue contained within the skull between these two regions was collected. The nasopharynx tissues were placed in 1.5 ml bead beating tubes, snap frozen in liquid nitrogen and stored at -80°C until metabolite extraction (for a period of no more than 3 months).

### Metabolite extraction

Metabolite extractions were carried out in a random order to ensure no batch effects. Each frozen sample was re-suspended in 0.8 ml ice-cold extraction solvent (50:50 [v/v]) acetonitrile/water. A bead beating protocol was used to homogenise the lung and nasopharynx tissues for metabolite extraction. For the lungs, 0.5 g of 2.8 mm diameter ceramic beads were added to tissues and, for the nasopharynx, 0.25 g of beads were used. Each tube was bead bashed for three minutes in total using a BeadBug tissue homogeniser (Sigma Aldrich, Gillingham, UK), in cycles of one minute with tubes put on ice between cycles. This process was carried out in a cold room, maintained between 2–4°C. After homogenisation the lysate was transferred to fresh 1.5 ml microfuge tubes and centrifuged at 4°C for ten minutes at 21,500 g. The supernatants were then transferred into fresh 1.5 ml microfuge tubes, snap frozen in liquid nitrogen and stored at -80°C until they were transferred on dry ice to University of Liverpool NMR Centre for lyophilisation and NMR profiling. The cell pellets were also frozen down and stored at -80°C in case further extraction of samples was required.

### NMR profiling

Supernatants from extracted samples were processed at the University of Liverpool NMR centre. These were lyophilised over-night at -55°C and processed before NMR sample preparation and NMR acquisition. NMR samples were resuspended in 200 μl 100 mM phosphate, pH 7.4 (100% ^2^H_2_O) and transferred to 3 mm outer diameter NMR tubes prior to acquisition on 700MHz Avance IIIHD spectrometer equipped with TCI cryoprobe (Bruker, Massachusetts, USA). Quality assurance of the spectrometer followed best practice [46,47] with temperature stability within 0.1°C using deuterated methanol thermometer [48] and 3 dimensional shiming on standard reference sample (2 mM sucrose), prior to sample acquisition, to ensure spectrometer optimisation. Samples were acquired using vendor supplier pulse sequence (cpmgpr1d) and automated acquisition and processing routines for maximum consistency.

Spectral acquisition was performed using standard ^1^H 1D Carr Purcell Meiboom Gill (CPMG) pulse sequence with 256 transients. All parameters are available with the deposited data in the EBI repository MetaboLights (MTBLS4591) [49]. Spectra were automatically processed with phasing, Fourier transformation and window function through vendor supplied routine (apk0.noe). Alignment was performed manually to Alanine CH3 doublet at 1.55 ppm.

### Data analysis

Spectra were binned interactively using an in house workflow within galaxy server toolkit tameNMR (https://github.com/PGB-LIV/tameNMR). Metabolites were matched to an in house library (supplemented with annotation from Chenomx v8.2 mammalian metabolite library). TopSpin (v4.1.3) was used to measure the peak boundaries to develop the nasopharynx and lung pattern files from a previous pattern file C2C12.

All statistical analysis was carried out using R statistical software (v4.1.0) with in house R scripts provided by the University of Liverpool computational biology facility (https://www.liverpool.ac.uk/computational-biology-facility) to perform established univariate and multivariate analyses [50,51].

Briefly, binned NMR spectrum datasets were normalised by probabilistic quotient normalisation (PQN) [52] before univariate analysis via one-way ANOVA with Bonferroni (Bonf) multiple testing correction and 0.05 significance level and Tukey’s simultaneous test for difference of means post hoc analysis. Fold changes were calculated with respect to the control or other test group and presented as natural log to indicate an increase (positive value) or decrease (negative value) of a given metabolite in a given group. Prior to in-depth analysis of data, we compared PQN to other methods of normalisation (tissue mass normalisation and total intensity normalisation) and found that the three methods produced comparable outputs.

Data were normalised by PQN and metabolite peaks scaled using the Pareto method, prior to multivariate analysis. Unsupervised Principal Component Analysis (PCA) was preformed to observe main sources of variance between samples and supervised partial least squares discriminant analysis (PLS-DA) was used to determine predictive models for metabolite profiles. PLS-DA plots were cross-validated against 30% randomly excluded samples to establish model quality as area under the receiver operating curve (AUROC) [53]. Ranked variable importance in projection (VIP) scores greater than 1 inferred which metabolites were influential for each PLS-DA model.

### Metabolite enrichment analysis

Metabolites of interest were mapped to pathways using metabolite set enrichment analysis (MSEA) within metaboanalyst (v5.0), using KEGG standard database supplied. The common compound name of metabolites with two or more significantly abundant peaks were input as lists into the metaboanalyst pathway analysis tool. The following parameters were selected ‐ the *Mus musculus* (mouse) pathway library, the Fishers’ exact test was used for the over-representation analysis and out degree centrality for the node importance measure for topological analysis.

### Bacterial growth analysis

Overnight cultures of D39 or D39_C20-3 in BHI were diluted to an optical density at 600 nm of 0.1 in 20% M17 media. From the same overnight cultures, separate dilutions to OD_600_ 0.1 were prepared in 20% M17 in which total BCAA concentration had been adjusted to 80 nM by addition of equal quantities of isoleucine, leucine and valine. Growth was measured in an automated plate reader (BMG LabTech), with OD600 readings taken every 15 minutes for 24 hours. Area under the logistic curve values were determined using the growthcurver package in R studio [54].

### qRT- PCR analysis of BCAA transport genes

Bacteria were inoculated into Brain Heart Infusion broth (Oxoid, Basingstoke, UK) and grown until mid-exponential phase of growth as determined by an OD_600_ reading of ~0.9. RNA was extracted from 1 ml of culture using the RiboPure RNA Purification Bacteria Kit (Invitrogen, California, USA) according to manufacturer’s instructions. RNA was then converted to cDNA using the iScript cDNA Synthesis Kit (Bio-Rad, California, USA). For qPCR, GoTaq qPCR Master Mix (Promega, Chilworth, UK) was used as per the manufacturer’s instructions. Triplicate reactions were set up using 2 μl of cDNA or nuclease free water (no template control) as a template and 0.2 μM of forward and reverse primers. Reactions were run on the CFX connect Real-Time PCR detection Thermocycler (Bio-Rad) under the following conditions: 2 min at 95°C followed by 40 cycles of 15 sec at 95°C and 1 min at 60°C. Gene expression changes were then assessed using the 2^-ΔΔCt^ method with *ddl* used as the housekeeping gene for normalisation of expression. Primer sequences were as follows; *codY_F* 5’-TCACCATGGCGGTCAATAC-3’, *codY_R* 5’-CAGAGCGAGTGATTCCGATAC-3’, *ddl_F* 5’-CCCAAGTTCCTTATGTGGCTATC-3’, *ddl_R* 5’-CCCATGTTTGACGGCTTAGT-3’, *livJ_F* 5’-ACTTCCAAGCAGCCCTTAC-3’, *livJ_R* 5’-CGCGCTTGGTTTACAATCTTAC-3’, *livG_F* 5’-TATGAACCAAGCGAGGGAAC-3’, *livG_R* 5’-AAGTACGTCCAAGTCCCAAAG-3’, *brnQ_F* 5’-TCAGTGGCCTTTAGCGTAATC-3’, *brnQ_R* 5’-CAACGATACCAACAACCCAAATAG-3’.

### Quantification of intracellular metabolite levels

For culture-based BCAA detection, bacterial strains were grown as described above. To assess intracellular metabolite levels, 1 ml of culture was pelleted to harvest bacterial cells. For detection in tissue, excised nasopharynx or lung tissue from naïve mice was homogenised in 2 ml PBS. Cells were lysed using 0.2% sodium deoxycholate (Sigma-Aldrich) and incubated at 37°C for 30 mins. To quantify intracellular BCAAs, 50 μl of lysate was transferred to a 96 well plate for use in the Abcam Branched Chain Amino Acids colourimetric kit (Abcam, Cambridge, UK). Assays were performed according to manufacturer’s instructions. Assay plates were incubated for 30 mins in the dark after which the OD of each well was measured at 450 nm using a BMG plate reader (BMG Labtech, Aylesbury, UK). Intracellular concentrations were normalised to culture density (Colony Forming Units, CFU) or tissue weight.

For quantification of intracellular phenylalanine levels, bacterial strains were grown as described above. Cells were cultured, pelleted and lysed, as above. For cell protein precipitation, 550 μl of lysate was mixed with 100 μl of ice cold perchloric acid (Sigma-Aldrich) and incubated on ice for 5 mins. Sample was neutralized by adding 20 μl of potassium hydroxide, gradually, to reach the pH range of 6.5 to 8.0. To quantify intracellular phenylalanine levels, 25 μl of lysate was transferred to a 96-well plate for use in the Abcam Phenylalanine Assay Kit (Abcam). Assays were performed according to manufacturer’s instructions. Assay plates were incubated in the dark at 37°C for 60 mins. Fluorescence was measured at (Ex/Em 535/587 nm) using a Varioskan microplate reader (Fisher Scientific, Loughborough, UK). Intracellular phenylalanine concentrations were normalised to culture density (CFU).

## Supporting information

S1 Table Genomic comparison of D39_C20-3 and D39. Nucleotide positions relative to the origin of replication are given. Mutation frequencies in the total D39_C20-3 population were identified from short-read sequence data, using Breseq2, with 100% indicating fixed mutations. Annotations show amino acid changes, and their corresponding codon position, alongside codon base pair changes. Δ indicates a deletion, + indicates an insertion. For intergenic mutations, annotation positions are relative to the nearest upstream and downstream genes. Syn = synonymous, SNP = single nucleotide polymorphism, del = deletion, ins = insertion. Data were originally reported in Green *et al* 2021 [20].
(DOCX)

S2 Table NMR pattern file for nasopharynx and lungs. Peak boundaries, peak numbers and associated metabolites are listed. Where a peak is unique to either lung or nasopharynx spectra, this is indicated by a + symbol. Notes are added to indicate small/margin peaks or where identified metabolites are from sample preparation carry-over.
(XLSX)

S1 Fig Pathway enrichment analysis of lung and nasopharynx NMR spectra. Pathway analysis was conducted using relative abundance of metabolites in lung vs nasopharynx, using MetaboAnalyst. Figures show enriched metabolic pathways in (**A**) lung and (**B**) nasopharynx samples. Nodes of importance with a p-value of ≤ 0.05 are labelled with the pathway name. The larger the circle, the higher the impact. Impact values consider the number of pathway metabolites that are differentially abundant, the magnitude of those differences, and the importance of individual differentially abundant metabolites within the overall pathway. Metabolites that are key intermediates (bottlenecks) in the pathway, or which connect to multiple other pathway metabolites are given higher impact scores. The colour of the circles, from red to yellow, denotes the significance, corresponding to the y-axis scale -log10(p).
(TIF)

S2 Fig Identification of enriched pathways in lung NMR data derived from naïve mice. Glutathione metabolism (**A**) and branched chain amino acid metabolism (**B**) were relatively enriched in lungs, as compared to nasopharynx. Analysis was conducted with MetaboAnalyst. Arrows show connectedness and directionality of connections between pathway intermediates. Metabolites in red are those found to be relatively more abundant in lung, as compared to nasopharynx. Where metabolite identification is unknown, KEGG identifiers are given.
(TIF)

S3 Fig Identification of enriched pathways in nasopharynx NMR data derived from naïve mice. The TCA cycle (**A**), arginine biosynthesis (**B**) and alpha amino acid pathways (**C**) were relatively enriched in nasopharynx, as compared to lungs. Analysis was conducted with MetaboAnalyst. Arrows show connectedness and directionality of connections between pathway intermediates. Metabolites in red are those found to be relatively more abundant in nasopharynx, as compared to the lungs. Where metabolite identification is unknown, KEGG identifiers are given.
(TIF)

S4 Fig Minimal changes in the nasopharyngeal metabolome during pneumococcal carriage. **A.** Principal component analysis of nasopharyngeal NMR data from sham-infected mice (PBS_Control) and those infected with D39 (Ancestor) or a nasopharynx-adapted D39 (C20-3). Relative abundance of metabolite peaks 386, associated with isoleucine (**B**) and 250, associated with tryptophan (**C**) in nasopharynx samples from D39_C20-3 infected mice, relative to PBS control mice or those infected with D39.
(JPG)

S5 Fig Changes in branched chain amino acid metabolism is a signature of adaptation to nasopharynx. **A.** Expression of four genes associated with branched chain amino acid synthesis, transport, or sensing, in nasopharynx-adapted D39 lineages, relative to the D39 ancestor from which they were derived. Expression levels were determined by qRT-PCR, using the 2^-ΔΔCt^ method. *ddl* was used as a housekeeping gene for normalisation of expression. Data analysis is by two-way ANOVA with Sidak’s multiple comparison test vs D39. n = 3 biological replicates per gene, per strain. Each biological replicate presented is the mean of 2–3 technical replicates. **B.** Branched chain amino acid (BCAA) and **C.** phenylalanine abundance in mid-log cultures of nasopharynx-adapted D39 and the D39 ancestor from which they were derived. Amino acid abundance was determined by colourimetric assay and normalised to culture density (colony forming units–CFU). Data are from 9 (D39, D39_C20-6, D39_C20-8) or 3 (D39_C20_1) independent cultures for BCAA and 4 independent cultures per strain for phenylalanine. Data analysis is by one-way ANOVA with Dunnett’s multiple comparison test vs D39. **D.** Colonisation potential of nasopharynx-adapted D39. Mice were administered 1 x 105 colony forming units of *S*. *pneumoniae* in 10 ul saline. Mice were sacrificed at 1, 7 or 14 days post-infection and infection burden determined in nasopharynx by tissue homogenisation and colony count. Data are from a single experiment, each data point represents an individual animal and p values are from two-way ANOVA with Dunnett’s post-test, with D39 as the comparator. * = p<0.05, ** = p<0.01, *** = p<0.001, **** = p<0.0001.
(TIF)

S1 Dataset PLS-DA models comparing infected and uninfected nasopharynx NMR spectra. (XLSX)

S2 Dataset PLS-DA models comparing infected and uninfected lung NMR spectra. (XLSX)
