# Supplementary material for: Airway metabolic profiling during Streptococcus pneumoniae infection identifies branched chain amino acids as signatures of upper airway colonisation
Source: PLoS Pathog. 2023 Sep 5;19(9):e1011630. doi: 10.1371/journal.ppat.1011630 (PMC10503754; doi:10.1371/journal.ppat.1011630)
Supplement: S1 Table — Nucleotide positions relative to the origin of replication are given. Mutation frequencies in the total D39_C20-3 population were identified from short-read sequence data, using Breseq2, with 100% indicating fixed mutations. Annotations show amino acid changes, and their corresponding codon position, alongside codon base pair changes. Δ indicates a deletion, + indicates an insertion. For intergenic mutations, annotation positions are relative to the nearest upstream and downstream genes. Syn = synonymous, SNP = single nucleotide polymorphism, del = deletion, ins = insertion. Data were originally reported in Green et al 2021 [20]. (DOCX) [file ppat.1011630.s001.docx]

| **Position** | **Mutation** | **Freq (%)** | **Annotation** | **Gene** | **Description** |
| --- | --- | --- | --- | --- | --- |
| 821,928 | Non-syn SNP | 100 | M122V (ATG→GTG) | *bceA* | Bacitracin export ATP‑binding protein |
| 1,888,307 | Non-syn SNP | 100 | G208R (GGA→AGA) | *gpsA* | Glycerol‑3‑phosphate dehydrogenase |
| 1,207,539 | Syn SNP | 100 | L155L (TTA→CTA) | *ltxB* | Leukotoxin export ATP‑binding protein |
| 1,066,272 | Non-syn SNP | 100 | P352R (CCC→CGC) | *phtA* | Histidine triad protein A |
| 1,321,986 | Non-syn SNP | 100 | S85A (TCA→GCA) | *D39N_01315* | hypothetical protein |
| 1,760,485 | Non-syn SNP | 100 | F370L (TTC→TTA) | *hlyX* | Putative hemolysin |
| 1,937,897 | Non-syn SNP | 100 | A608S (GCA→TCA) | *D39N_01974* | hypothetical protein |
| 1,121,524 | Intergenic SNP | 100 | G→T (+12/‑270) | *panT/niaX* |  |
| 1,574,264 | Intergenic del | 100 | Δ1 bp (‑59/+132) |  |  |
| 1,522,456 | Intergenic ins | 100 | +T (‑581/+434) |  |  |
| 702,134 | Non-syn SNP | 100 | S331N (AGC→AAC) | *yknX* | Putative efflux system component |
| 1,626,115 | Intergenic SNP | 94 | C→A (‑12/+13) |  |  |
| 456,001 | Intergenic SNP | 65 | C→T (+16/‑61) |  |  |
| 458,532 | Intergenic SNP | 56 | C→T (+402/‑49) |  |  |
| 462,626 | Syn SNP | 49 | S74S (AGT→AGC) | *hsdS_2* | Type‑1 restriction modification system |
| 564,899 | Non-syn SNP | 40 | E72V (GAA→GTA) | *D39N_00558* | hypothetical protein |
| 660,806 | Intergenic SNP | 30 | G→A (‑178/‑255) |  |  |
| 660,805 | Intergenic SNP | 30 | G→A (‑177/‑256) |  |  |
| 660,832 | Intergenic SNP | 22 | G→A (‑204/‑229) |  |  |
| 808,358 | Non-syn SNP | 21 | F57L (TTC→TTA) | *pyk* | Pyruvate kinase |
| 660,515 | Syn SNP | 12 | A38A (GCT→GCC) | *D39N_00652* | hypothetical protein |
| 660,856 | Intergenic SNP | 11 | G→A (‑228/‑205) |  |  |
| 577,366 | Intergenic SNP | 7 | G→A (+570/‑55) |  |  |
