# Supplementary figures and images for: Airway metabolic profiling during Streptococcus pneumoniae infection identifies branched chain amino acids as signatures of upper airway colonisation
Source: PLoS Pathog. 2023 Sep 5;19(9):e1011630. doi: 10.1371/journal.ppat.1011630 (PMC10503754; doi:10.1371/journal.ppat.1011630)

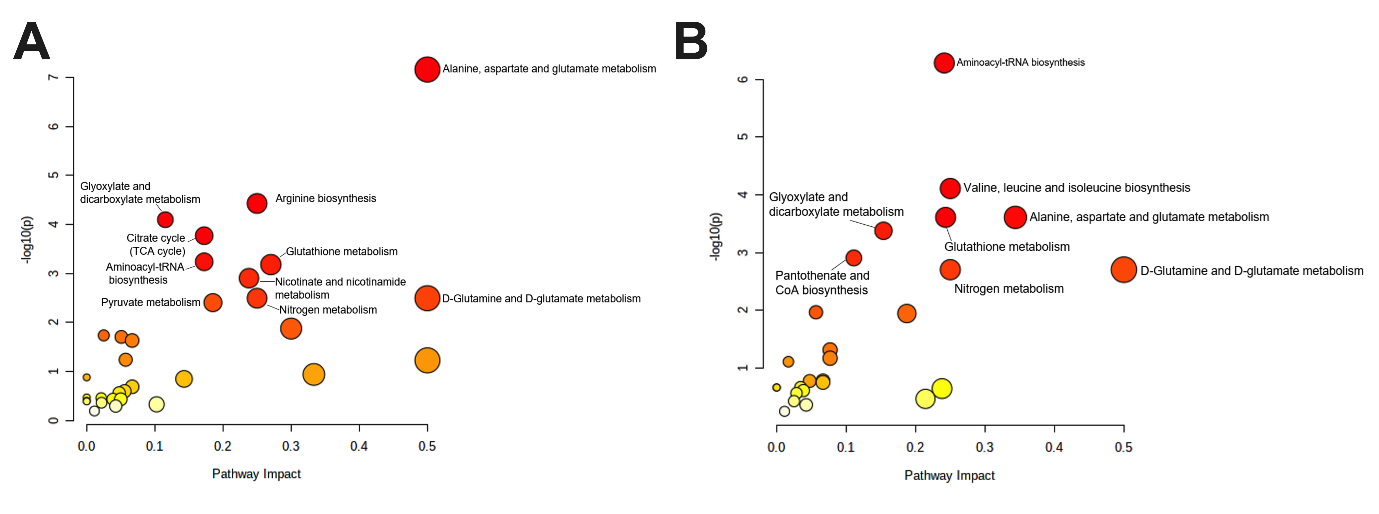

Supplement: S1 Fig — Pathway analysis was conducted using relative abundance of metabolites in lung vs nasopharynx, using MetaboAnalyst. Figures show enriched metabolic pathways in (A) lung and (B) nasopharynx samples. Nodes of importance with a p-value of ≤ 0.05 are labelled with the pathway name. The larger the circle, the higher the impact. Impact values consider the number of pathway metabolites that are differentially abundant, the magnitude of those differences, and the importance of individual differentially abundant metabolites within the overall pathway. Metabolites that are key intermediates (bottlenecks) in the pathway, or which connect to multiple other pathway metabolites are given higher impact scores. The colour of the circles, from red to yellow, denotes the significance, corresponding to the y-axis scale -log10(p). (TIF) [file ppat.1011630.s003.tif]

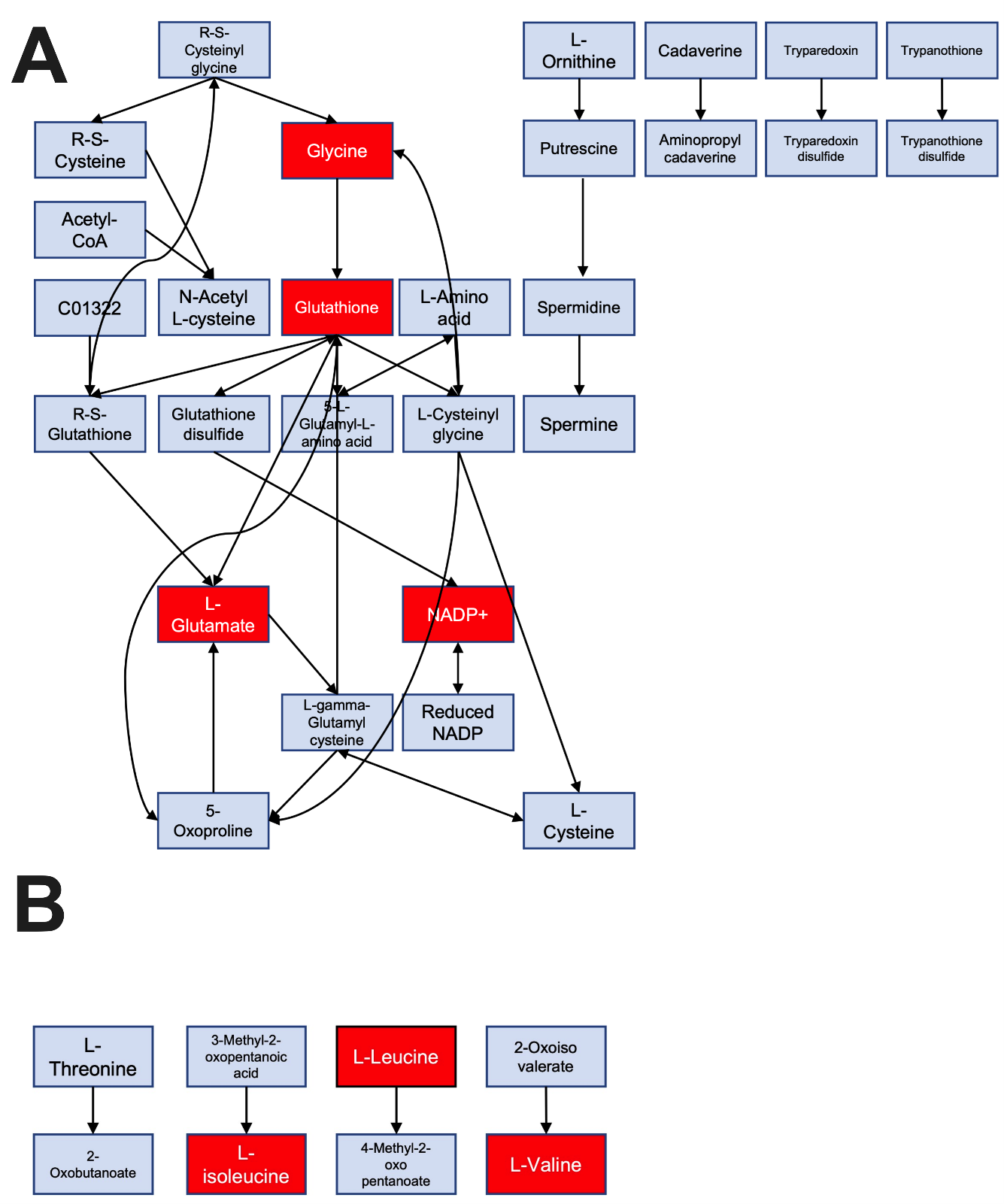

Supplement: S2 Fig — Glutathione metabolism (A) and branched chain amino acid metabolism (B) were relatively enriched in lungs, as compared to nasopharynx. Analysis was conducted with MetaboAnalyst. Arrows show connectedness and directionality of connections between pathway intermediates. Metabolites in red are those found to be relatively more abundant in lung, as compared to nasopharynx. Where metabolite identification is unknown, KEGG identifiers are given. (TIF) [file ppat.1011630.s004.tif]

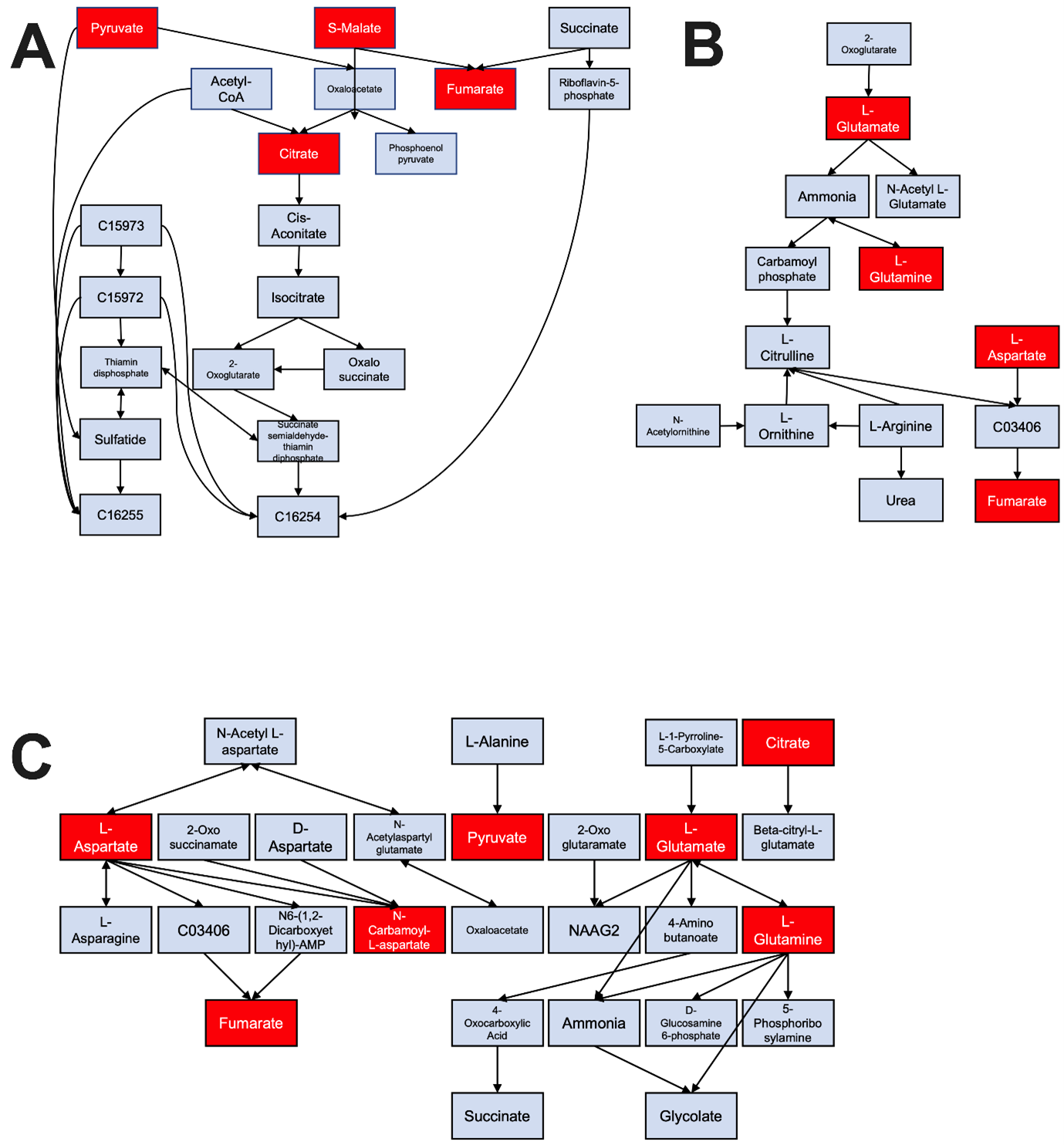

Supplement: S3 Fig — The TCA cycle (A), arginine biosynthesis (B) and alpha amino acid pathways (C) were relatively enriched in nasopharynx, as compared to lungs. Analysis was conducted with MetaboAnalyst. Arrows show connectedness and directionality of connections between pathway intermediates. Metabolites in red are those found to be relatively more abundant in nasopharynx, as compared to the lungs. Where metabolite identification is unknown, KEGG identifiers are given. (TIF) [file ppat.1011630.s005.tif]

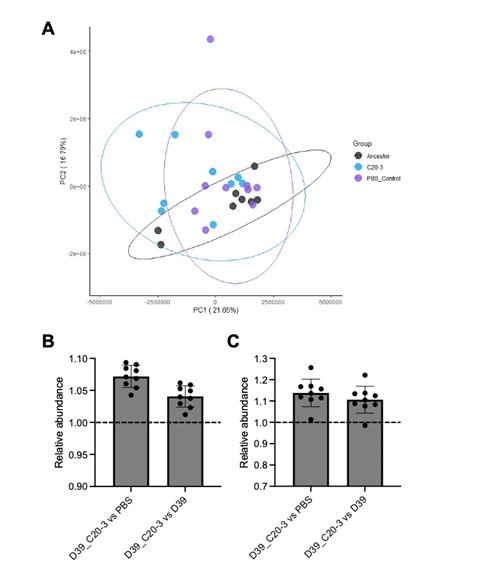

Supplement: S4 Fig — A. Principal component analysis of nasopharyngeal NMR data from sham-infected mice (PBS_Control) and those infected with D39 (Ancestor) or a nasopharynx-adapted D39 (C20-3). Relative abundance of metabolite peaks 386, associated with isoleucine (B) and 250, associated with tryptophan (C) in nasopharynx samples from D39_C20-3 infected mice, relative to PBS control mice or those infected with D39. (JPG) [file ppat.1011630.s006.jpg]

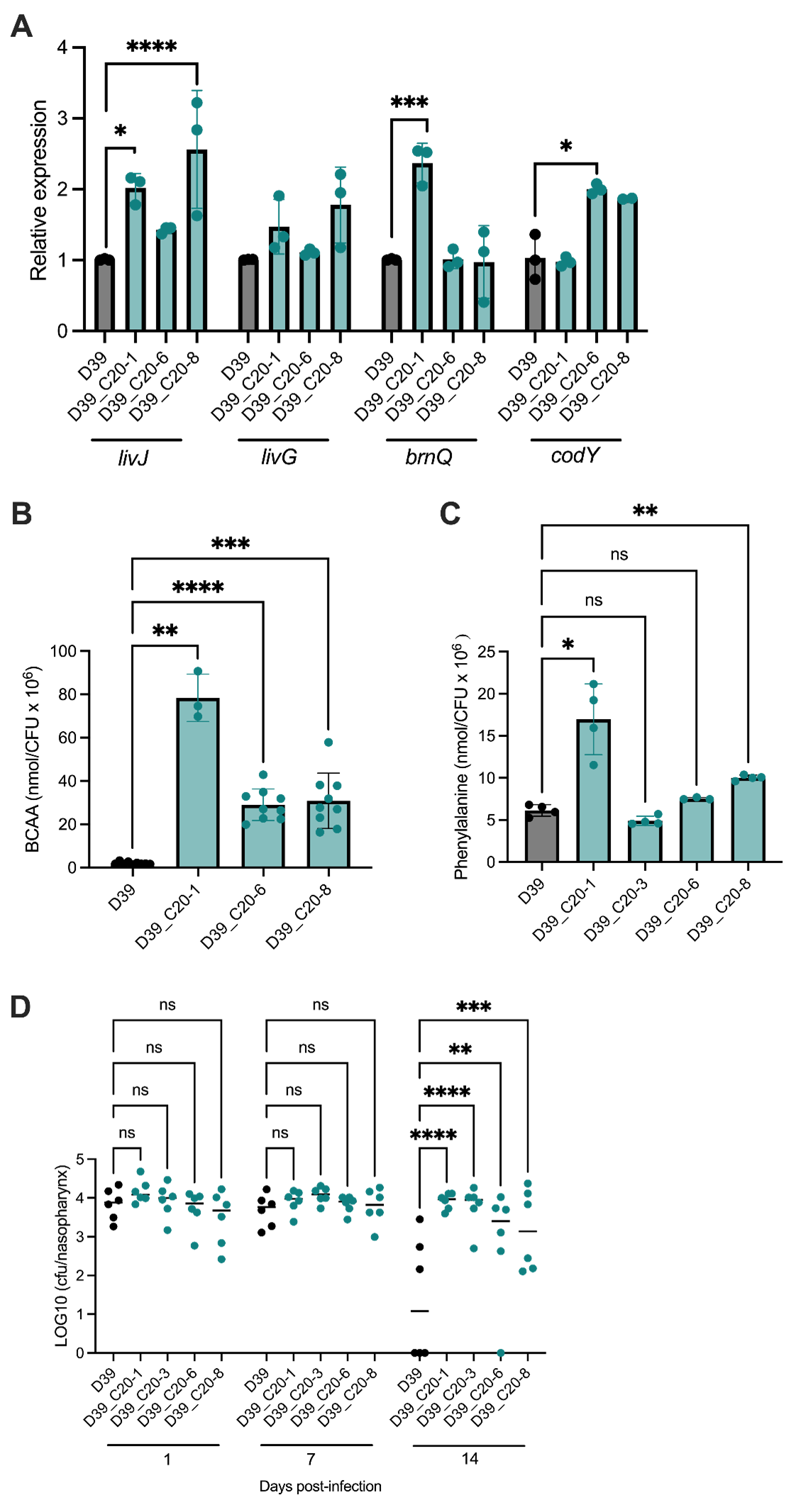

Supplement: S5 Fig — A. Expression of four genes associated with branched chain amino acid synthesis, transport, or sensing, in nasopharynx-adapted D39 lineages, relative to the D39 ancestor from which they were derived. Expression levels were determined by qRT-PCR, using the 2-ΔΔCt method. ddl was used as a housekeeping gene for normalisation of expression. Data analysis is by two-way ANOVA with Sidak’s multiple comparison test vs D39. n = 3 biological replicates per gene, per strain. Each biological replicate presented is the mean of 2–3 technical replicates. B. Branched chain amino acid (BCAA) and C. phenylalanine abundance in mid-log cultures of nasopharynx-adapted D39 and the D39 ancestor from which they were derived. Amino acid abundance was determined by colourimetric assay and normalised to culture density (colony forming units–CFU). Data are from 9 (D39, D39_C20-6, D39_C20-8) or 3 (D39_C20_1) independent cultures for BCAA and 4 independent cultures per strain for phenylalanine. Data analysis is by one-way ANOVA with Dunnett’s multiple comparison test vs D39. D. Colonisation potential of nasopharynx-adapted D39. Mice were administered 1 x 10^5^ colony forming units of S. pneumoniae in 10 ul saline. Mice were sacrificed at 1, 7 or 14 days post-infection and infection burden determined in nasopharynx by tissue homogenisation and colony count. Data are from a single experiment, each data point represents an individual animal and p values are from two-way ANOVA with Dunnett’s post-test, with D39 as the comparator. * = p<0.05, ** = p<0.01, *** = p<0.001, **** = p<0.0001. (TIF) [file ppat.1011630.s007.tif]
